# Synanthropic Plants as an Underestimated Source of Bioactive Phytochemicals: A Case of *Galeopsis bifida* (Lamiaceae)

**DOI:** 10.3390/plants9111555

**Published:** 2020-11-12

**Authors:** Daniil N. Olennikov

**Affiliations:** Laboratory of Medical and Biological Research, Institute of General and Experimental Biology, Siberian Division, Russian Academy of Science, 6 Sakhyanovoy Street, Ulan-Ude 670047, Russia; olennikovdn@mail.ru; Tel.: +7-9021-600-627

**Keywords:** *Galeopsis bifida*, Lamiaceae, phenylethanoid glycosides, flavone glycosides, HPLC, mass-spectrometry, chemotypes, population, organ-specificity, antioxidant activity

## Abstract

Hemp nettle (*Galeopsis bifida* Boenn.) is a synanthropic species of the Lamiaceae family that is widely distributed across Europe, Asia, and Siberia. *Galeopsis bifida* is deeply embedded in the ethnomedical tradition of Asian healers; however, this plant is still poorly characterized, both chemically and pharmacologically. To study Siberian populations of *G. bifida*, we used high-performance liquid chromatography with photodiode array and electrospray triple quadrupole mass detection for metabolic profiling. Ninety compounds were identified, including iridoid glycosides, phenylethanoid glycosides, hydroxycinnamates, and flavone glycosides, most of which were identified in *G. bifida* for the first time, while some phenolics were found to have potential chemotaxonomic significance in the Lamiaceae family and *Galeopsis* genus. An unequal quantitative distribution of the selected metabolites was observed within separate organs of the *G. bifida* plant, characterized by high accumulation of most compounds within the aerial part of the plant (leaves, flowers). Analysis of the content of specific chosen compounds within the leaves of different populations of *G. bifida* from Eastern Siberia revealed the existence of two chemical types based on metabolic specifics: the southern type accumulates flavone glucuronides, while the northern type tends to accumulate high levels of phenylpropanoids and acylated flavone glucosides. The first study of the bioactivity of *G. bifida* extract demonstrated that the herb has low toxicity in acute experiments and expresses antioxidant potential against free radicals in the form of DPPH˙, ABTS˙^+^, and superoxide radical, as well as high ferric reducing antioxidant power, oxygen radical absorbance capacity, and protective action in the carotene bleaching assay. In general, our results suggest the herb of *G. bifida* as a new, prospective synanthropic plant for medical application.

## 1. Introduction

Intensive use of natural landscapes and decreased areas of natural vegetation lead to the process of synanthropization, which has acquired the scale of anthropogenic evolution [1]. In a broad sense, synathropization refers to the process of adaptation of organisms to habitats in places dramatically transformed by humans, up to settlements and human dwellings. In connection with anthropogenic transformation, synanthropic species occupy an increasingly prominent place in the structure of biological diversity, which is especially important for the vast territories of Siberia and Asia [2]. Speaking of their practical importance, synanthropic species, as a rule, are not considered to be economically valuable due to the instability of their raw material base. However, these species are characterized by high reproductive energy as well as wide ranging ecological adaptability, which make these species convenient for introduction into culture [3]. The study of prospective practical applications for synanthropic species will, in the future, solve the problem of their utilization and expand the range of useful plant species.

Among the various synanthropic species of Eurasia, the weed genus *Galeopsis* L. of the Lamiaceae family is currently represented by 14 species (10 of which grow in Eurasia) belonging to two subgenera, *Galeopsis* (*Tetrahit*) and *Ladanum* [4,5]. Botanically, *Galeopsis* species are annual pubescent grasses with ovoid or lanceolate petiolate leaves and sessile flowers in whorls in the leaf axils. These species are often found in crops, fields, garbage sites, and alongside roads. Information on the chemical composition of *Galeopsis* species indicates the presence of iridoids [6,7,8,9,10,11], diterpenes [12,13,14], triterpenes [13], benzoic acids [15], hydroxycinnamates [15,16], flavones [9,15,17,18,19,20,21], fatty acids [22,23,24,25], acylglycerols [26], and essential oils [27,28] (Table 1). Early pharmacological studies have indicated the presence of central nervous system (CNS) depressive [29], antioxidant [30], neuroprotective, and anticholinesterase activities [31] in extracts of the *Galeopsis* species.

Regarding the territory of Siberia, the most abundant synanthropic species is *G. bifida* Boenn. (hemp nettle) [32,33], which, despite its large biological reserves, has no economic value, although it is often used in various traditional medical systems of the Siberian peoples and some Asian countries. In Tibetan medicine, as well as its local branch in the form of Buryat traditional medicine, the herb *G. bifida* is widely used under various names (*‘jib rtsi*, *pri yang ky*, *zhim thig le*) in the form of decoctions, rinses, and irrigation solutions, as well as applied in the treatment of various oral diseases (stomatitis, caries), gastrointestinal tract disorders (gastritis, gastroenteritis, ulcers, and inflammation of the esophagus, stomach, and intestines), kidney disorders (inflammation, cystitis), inflammation of the lungs and female genital organs, and eye diseases (conjunctivitis) [34,35]. In the medicine of the peoples of the Far East, infusions of *G. bifida* in vodka have been used in the treatment of oncological diseases of the stomach, sore throat, and epilepsy, as well as to increase food bitterness to stimulate appetite [36]. Additionally, leaf applications of *G. bifida* have been used to treat lichen, panaritium, and other skin wounds [36]. In the territory of Siberia, a decoction of the plant has been used as an expectorant to treat pulmonary tuberculosis and other respiratory infections, while a milk infusion was used for chronic rhinitis [37]. In the medical practice of the nomadic peoples of the North, a tincture of *G. bifida* herb was used to treat liver diseases [38]. In Kyrgyzstan, *G. bifida* tincture was recommended as an antihypertensive agent [39]. It should also be noted that young leaves of *G. bifida* were used earlier in the Baikal region, and are still used there today in food as a salad plant [40].

An ambiguous opinion exists regarding the toxicity of *G. bifida*, as well as *Galeopsis* species in general. Scientific reports [41] indicate that there is a possibility of temporary paralysis of the limbs when eating oil from the seeds of some species of *Galeopsis* (*G. bifida*, *G. ladanum*, *G. speciosa*, and *G. tetrahit*). A case of oil poisoning by seeds of *G. segetum* (also known as *G. cannabina*) has been described, which resulted in nausea, a feeling of heaviness in the lower extremities, and pain in the hands and in the region of the sacrum [42]. A scientific study of this phenomenon has not been carried out; therefore, the question of the reliability of this information remains open. However, a more recent study investigating the chemical causes of a pathological condition known as koturnism, which is caused by consumption of the meat of some species of quail that feed on *G. ladanum* [43], did not observe any toxic manifestations with regards to *G. ladanum* extract or stachydrin, which is its component.

Currently, despite satisfactory raw material reserves, *G. bifida* does not have any practical application, for example, as an official medicinal plant. The reason for this is lack of knowledge regarding the plant’s chemical composition, as well as the lack of information on the pharmacological effects of extraction preparations from it. The known data on the metabolites of *G. bifida* indicate the presence of iridoids [7,8], flavonoids [17], fatty acids [22,23,24,25], acylglycerols [26], and essential oils [27].

In the current study, we performed a qualitative chromatographic analysis of *G. bifida* using high-performance liquid chromatography with photodiode array and electrospray triple quadrupole mass detection (HPLC-PAD-ESI-tQ-MS). Additionally, we performed quantification of selected metabolites within the different organs of *G. bifida* and natural populations, as well as investigations into the acute toxicity and antioxidant properties of the plant using various biological in vitro assays.

## 2. Results and Discussion

### 2.1. Metabolites of Galeopsis bifida: LC-MS Profile and Chemotaxonomic Significance

**Figure 1:**
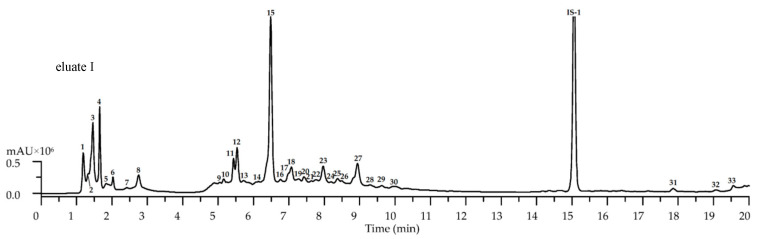

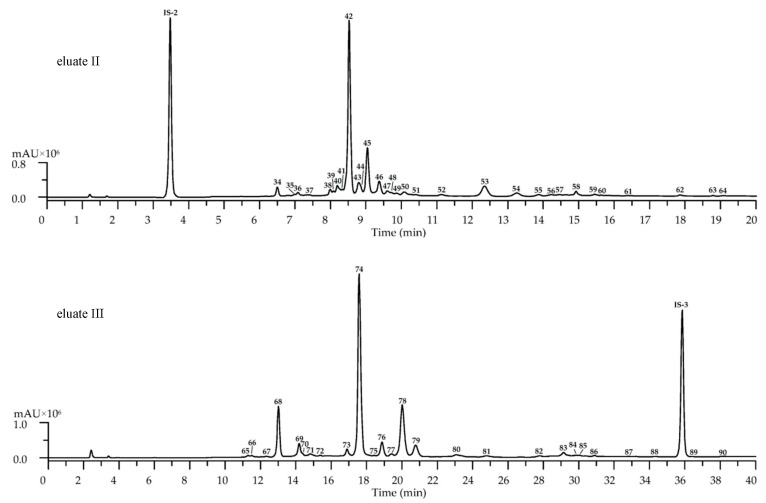
High-performance liquid chromatography with electrospray ionization triple quadrupole mass spectrometric detection (HPLC-ESI-tQ-MS) chromatogram (base peak chromatogram or BPC mode) of solid-phase extraction (SPE)-polyamide eluate I (chromatographic conditions—mode 1, positive ionization), eluate II (chromatographic conditions—mode 2, negative ionization), and eluate III (chromatographic conditions—mode 3, negative ionization) of *Galeopsis bifida* herb extract. Compounds are numbered as listed in Table 2. Internal standards: IS-1—trifloroside (38 μg/mL); IS-2—scopoletin-7-*O*-neohesperidoside (48 μg/mL); IS-3—3,5-di-*O*-feruloylquinic acid (9.5 μg/mL).

The existing data regarding the metabolites of *Galeopsis* species indicate the presence of a wide group of compounds with various polarities and chromatographic behaviors (Table 1). Therefore, prior to studying the LC-MS profile of *G. bifida* extract, we separated the total probe using a solid-phase extraction (SPE) technique on a polyamide cartridge in order to avoid missing any low content or trace compounds. Elution of the preconditioned cartridge using water, ethanol, and alkalized ethanol allowed the isolatation of a hydrophilic fraction of iridoids and two less polar fractions of phenylethanoid glycosides/neutral flavone glycosides and acidic/acylated flavone glycosides. Further analysis of the three eluates by HPLC-PAD-ESI-tQ-MS assay (Figure 1) demonstrated the good separation of a total of ninety compounds, identified by their retention times and ultraviolet (UV) and mass spectrometric data via comparison with reference standards and known literature information (Table 2).

#### 2.1.1. Iridoid Glycosides

Iridoid glycosides are highly hydrophilic compounds that showed good eluatability after water elution of the SPE-polyamide cartridge. Thirty-three compounds (**1**–**33**) were detected after HPLC-PAD-ESI-tQ-MS separation. Six iridoid glycosides were identified after comparison with reference standards, comprising harpagide (**3**), harpagide 8-*O*-acetate (**15**), ajugol (**20**), secologanin (**23**), reptoside (**25**), and ajugoside (**27**). Compounds **3** and **15** are common components of *Galeopsis* species [8,9] and the Lamiaceae family [44]. Ajugoside has previously been identified in *G. bifida* [7], while compounds **20**, **23**, and **25** were detected in the species for the first time in the current study. Of the other iridoid glycosides, harpagide derivatives predominated, accounting for 15 of the 33 compounds (**1**, **5**, **9**, **10**, **13**, **14**, **18**, **19**, **21**, **22**, **24**, **26**, and **28**–**30**). The mass spectrometric data showed that the protonated ions [M+H]^+^ can lose the various number of hexose (−162 a.m.u.) and/or acetyl fragments (−42 a.m.u.) [45,46] necessary to identify the unknown compounds as harpagide *O*-hexoside (**1**), harpagide *O*-acetyl-*O*-hexoside (**9**, **10**), harpagide di-*O*-acetyl-*O*-hexoside (**13**, **14**), harpagide tri-*O*-acetyl-di-*O*-hexoside (**18**, **19**), harpagide di-*O*-acetate (**21**, **22**), harpagide tri-*O*-acetyl-*O*-hexoside (**24**, **26**), or harpagide tri-*O*-acetate (**28**–**30**).

**Table 2:** Retention times (*t*_R_), UV- and ESI-MS spectral data of compounds **1**–**90** found in *Galeopsis bifida*.

**No**	***t_R_*** **(min) ^a^**	**Compound ^b^**	**UV Pattern ^c^**	**CE (eV) ^d^**	**ESI-MS (*m*/*z*)**		
					**MS ^e^**	**MS/MS (*I*, %) ^f^**	**Adducts ^g^**
**Positive ionization**							
**1**	1.20 ^i^	Harpagide *O*-Hex ^L^ [45,46]	IG	+25	527	[527]: 365 (100), 351 (11), 203 (15)	549 ^Na^
**2**	1.35 ^i^	Galiridoside *O*-Hex ^L^ [46,47]	IG	+25	509	[509]: 347 (100), 331 (9), 185 (17)	531 ^Na^
**3**	1.48 ^i^	Harpagide ^S^ [45]	IG	+20	365	[365]: 351 (5), 203 (100)	387 ^Na^
**4**	1.69 ^i^	Galiridoside ^L^ [47]	IG	+20	347	[347]: 331 (2), 185 (100)	369 ^Na^
**5**	1.84 ^i^	Desoxy-harpagide ^L^ [48]	IG	+20	349	[349]: 187 (100)	371 ^Na^
**6**	2.02 ^i^	Antirrhinoside *O*-Hex ^L^ [46,49]	IG	+25	525	[525]: 363 (100), 347 (4), 201 (10)	547 ^Na^
**7**	2.43 ^i^	Galiridoside *O*-Ac ^L^ [46,47]	IG	+25	389	[389]: 247 (5), 227 (100), 185 (25)	411 ^Na^
**8**	2.73 ^i^	Antirrhinoside ^L^ [49]	IG	+20	363	[363]: 347 (8), 201 (100)	385 ^Na^
**9**	5.04 ^i^	Harpagide *O*-Ac-*O*-Hex ^L^ [45,46]	IG	+15	569	[569]: 407 (100) [407]: 365 (12), 245 (100), 203 (19)	591 ^Na^
**10**	5.12 ^i^	Harpagide *O*-Ac-*O*-Hex ^L^ [45,46]	IG	+20	569	[569]: 407 (100) [407]: 365 (14), 245 (100), 203 (24)	591 ^Na^
**11**	5.42 ^i^	Antirrhinoside *O*-Ac ^L^ [46,49]	IG	+25	405	[405]: 363 (11), 243 (100), 201 (22)	427 ^Na^
**12**	5.56 ^i^	Antirrhinoside *O*-Ac ^L^ [46,49]	IG	+25	405	[405]: 363 (9), 243 (100), 201 (20)	427 ^Na^
**13**	5.68 ^i^	Harpagide *O*-Ac_2_-*O*-Hex ^L^ [45,46]	IG	+20	611	[611]: 569 (23), 407 (100) [407]: 365 (12), 245 (100), 203 (20)	633 ^Na^
**14**	6.14 ^i^	Harpagide *O*-Ac_2_-*O*-Hex ^L^ [45,46]	IG	+20	611	[611]: 569 (22), 407 (100) [407]: 365 (9), 245 (100), 203 (25)	633 ^Na^
**15**	6.48 ^i^	Harpagide 8-*O*-Ac ^S^ [45,46]	IG	+20	407	[407]: 365 (15), 245 (100), 203 (24)	429 ^Na^
**16**	6.78 ^i^	Reptoside or ajugoside *O*-Hex ^L^ [46,48,50]	IG	+22	553	[553]: 319 (100) [391]: 349 (6), 229 (100), 187 (19)	575 ^Na^
**17**	6.97 ^i^	Reptoside or ajugoside *O*-Hex ^L^ [46,48,50]	IG	+22	553	[553]: 319 (100) [391]: 349 (16), 229 (100), 187 (19)	575 ^Na^
**18**	7.12 ^i^	Harpagide *O*-Ac_3_-*O*-Hex_2_^L^ [45,46]	IG	+22	815	[815]: 653 (100), 491 (54) [491]: 449 (7), 407 (12), 245 (100) [407]: 365 (9), 245 (100), 203 (17)	837 ^Na^
**19**	7.26 ^i^	Harpagide *O*-Ac_3_-*O*-Hex_2_^L^ [45,46]	IG	+22	815	[815]: 653 (100), 491 (50) [491]: 449 (5), 407 (16), 245 (100) [407]: 365 (10), 245 (100), 203 (21)	837 ^Na^
**20**	7.45 ^i^	Ajugol ^S^ [50]	IG	+25	349	[349]: 187 (100), 171 (5)	371 ^Na^
**21**	7.68 ^i^	Harpagide *O*-Ac_2_ [45,46]	IG	+25	449	[449]: 407 (100), 245 (29) [407]: 365 (8), 245 (100), 203 (10)	471 ^Na^
**22**	7.79 ^i^	Harpagide *O*-Ac_2_^L^ [45,46]	IG	+25	449	[449]: 407 (100), 245 (25) [407]: 365 (10), 245 (100), 203 (15)	471 ^Na^
**23**	7.98 ^i^	Secologanin ^S^ [46]	IG	+28	389	[389]: 375 (11), 227 (100), 213 (8)	411 ^Na^
**24**	8.18 ^i^	Harpagide *O*-Ac_3_-*O*-Hex ^L^ [45,46]	IG	+22	653	[653]: 491 (100) [491]: 449 (6), 407 (10), 245 (100) [407]: 365 (10), 245 (100), 203 (15)	675 ^Na^
**25**	8.33 ^i^	Reptoside ^S^ [48]	IG	+25	391	[391]: 349 (4), 229 (100), 187 (17)	413 ^Na^
**26**	8.49 ^i^	Harpagide *O*-Ac_3_-*O*-Hex ^L^ [45,46]	IG	+22	653	[653]: 491 (100) [491]: 449 (4), 407 (11), 245 (100) [407]: 365 (12), 245 (100), 203 (14)	675 ^Na^
**27**	8.97 ^i^	Ajugoside ^S^ [50]	IG	+ 25	391	[391]: 349 (15), 229 (100), 187 (15)	413 ^Na^
**28**	9.29 ^i^	Harpagide *O*-Ac_3_^L^ [45,46]	IG	+25	491	[491]: 449 (3), 407 (12), 245 (100) [407]: 365 (10), 245 (100), 203 (12)	513 ^Na^
**29**	8.67 ^i^	Harpagide *O*-Ac_3_^L^ [45,46]	IG	+25	491	[491]: 449 (5), 407 (10), 245 (100) [407]: 365 (8), 245 (100), 203 (7)	513 ^Na^
**30**	10.01 ^i^	Harpagide *O*-Ac_3_^L^ [45,46]	IG	+25	491	[491]: 449 (3), 407 (14), 245 (100) [407]: 365 (4), 245 (100), 203 (14)	513 ^Na^
**31**	17.85 ^i^	Reptoside or ajugoside *O*-Ac ^S^ [46,48,50]	IG	+25	433	[433]: 391 (9), 229 (100) [391]: 349 (27), 229 (100), 187 (14)	455 ^Na^
**32**	19.09 ^i^	Reptoside or ajugoside *O*-Ac ^L^ [46,48,50]	IG	+25	433	[433]: 391 (26), 229 (100) [391]: 349 (14), 229 (100), 187 (17)	455 ^Na^
**33**	19.57 ^i^	Reptoside or ajugoside *O*-Ac ^L^ [46,48,50]	IG	+25	433	[433]: 391 (21), 229 (100) [391]: 349 (11), 229 (100), 187 (15)	455 ^Na^
**Negative ionization**							
**34**	6.48 ^ii^	Verbascoside/ isoverbascoside *O*-Pent_2_^L^ [51]	PEG	−30	887	[887]: 755 (100), 593 (20) [593]: 461 (100), 315 (24), 153 (4)	
**35**	7.02 ^ii^	Verbascoside/ isoverbascoside *O*-Pent_2_^L^ [51]	PEG	−30	887	[887]: 755 (69), 623 (100), 491 (11), 461 (26) [461]: 315 (100), 153 (27)	
**36**	7.09 ^ii^	Verbascoside/ isoverbascoside *O*-Pent_2_^L^ [51]	PEG	−30	887	[887]: 755 (73), 623 (100), 491 (15), 461 (19) [461]: 315 (100), 153 (16)	
**37**	7.38 ^ii^	Hydroxy-verbascoside ^L^ [51]	PEG	−25	639	[639]: 493 (15), 477 (100), 331 (12) [477]: 331 (100), 169 (14)	685 ^FA^
**38**	7.97 ^ii^	Lavandulifolioside ^S^ [51,52]	PEG	−25	755	[755]: 623 (5), 603 (1), 593 (100), 461 (45) [461]: 315 (100), 153 (26)	801 ^FA^
**39**	8.11 ^ii^	Leucosceptoside A *O*-Pent_2_^L^ [51,52]	PEG	−25	901	[901]: 769 (63), 637 (100) [637]: 505 (10), 461 (100) [461]: 315 (100), 153 (9)	
**40**	8.23 ^ii^	Verbascoside/ isoverbascoside *O*-Pent ^L^ [51]	PEG	−30	755	[755]: 623 (11), 603 (2), 593 (100), 461 (40) [461]: 315 (100), 153 (14)	801 ^FA^
**41**	8.33 ^ii^	Leucosceptoside A *O*-Pent ^L^ [51,52]	PEG	−25	769	[769]: 637 (11), 617 (1), 593 (100), 461 (14) [461]: 315 (100), 153 (18)	815 ^FA^
**42**	8.51 ^ii^	Verbascoside ^S^ [51]	PEG	−25	623	[623]: 477 (2), 471 (1), 461 (100), 161 (23) [461]: 315 (100), 153 (31)	669 ^FA^
**43**	8.77 ^ii^	Leonoside A ^S^ [51]	PEG	−25	769	[769]: 637 (9), 617 (2), 593 (100), 461 (27) [461]: 315 (100), 153 (30)	815 ^FA^
**44**	8.92 ^ii^	Luteolin 7-*O*-Glc ^S^ [53]	LG	−20	447	[447]: 285 (100)	493 ^FA^
**45**	9.06 ^ii^	Isoverbascoside ^S^ [51]	PEG	−25	623	[623]: 477 (1), 471 (1), 461 (100), 161 (19) [461]: 315 (100), 153 (26)	669 ^FA^
**46**	9.43 ^ii^	Leucosceptoside A ^S^ [51,52]	PEG	−25	637	[637]: 505 (12), 461 (100) [461]: 315 (100), 153 (12)	683 ^FA^
**47**	9.56 ^ii^	Leonoside B ^S^ [51]	PEG	−27	783	[783]: 651 (5), 607 (100), 475 (14) [607]: 475 (100), 329 (32), 167 (14)	829 ^FA^
**48**	9.72 ^ii^	Verbascoside/ isoverbascoside *O*-Pent_2_-*O*-Caf ^L^ [46,51]	PEG	−35	1049	[1049]: 887 (62), 755 (32), 623 (100) [623]: 477 (5), 471 (2), 461 (100), 161 (14) [461]: 315 (100), 153 (29)	
**49**	9.92 ^ii^	Verbascoside/ isoverbascoside *O*-Pent_2_-*O*-Caf ^L^ [46,51]	PEG	−35	1049	[1049]: 887 (54), 755 (52), 623 (100) [623]: 477 (3), 471 (1), 461 (100), 161 (19) [461]: 315 (100), 153 (25)	
**50**	10.12 ^ii^	Apigenin 7-*O*-Glc ^S^ [53]	AG	−20	431	[431]: 269 (100)	477 ^FA^
**51**	10.45 ^ii^	Verbascoside/ isoverbascoside *O*-Pent_2_-*O*-Caf ^L^ [46,51]	PEG	−35	1049	[1049]: 887 (52), 755 (63), 623 (100) [623]: 477 (1), 471 (1), 461 (100), 161 (26) [461]: 315 (100), 153 (18)	
**52**	11.15 ^ii^	Martynoside ^S^ [52]	PEG	−30	651	[651]: 505 (2), 475 (100) [475]: 329 (100), 167 (29)	697 ^FA^
**53**	12.41 ^ii^	Verbascoside/ isoverbascoside *O*-Pent-*O*-Caf ^L^ [46,51]	PEG	−35	917	[917]: 755 (48), 623 (100), 461 (11) [623]: 477 (5), 471 (1), 461 (100), 161 (14) [461]: 315 (100), 153 (26)	
**54**	13.25 ^ii^	Verbascoside/ isoverbascoside *O*-Pent-*O*-Caf ^L^ [46,51]	PEG	−35	917	[917]: 755 (36), 623 (100), 461 (15) [623]: 477 (3), 471 (1), 461 (100), 161 (18) [461]: 315 (100), 153 (14)	
**55**	13.90 ^ii^	Leucosceptoside A *O*-Pent-*O*-Caf ^L^ [46,51]	PEG	−30	931	[931]: 769 (89), 637 (100) [637]: 505 (16), 461 (100) [461]: 315 (100), 153 (14)	
**56**	14.22 ^ii^	Leucosceptoside A *O*-Pent-*O*-Caf ^L^ [46,51]	PEG	−30	931	[931]: 769 (73), 637 (100) [637]: 505 (15), 461 (100) [461]: 315 (100), 153 (9)	
**57**	14.48 ^ii^	Leonoside B *O*-Pent-*O*-Caf ^L^ [46,51]	PEG	−35	1077	[1077]: 915 (73), 783 (100) [783]: 651 (7), 607 (100), 475 (12) [607]: 475 (100), 329 (30), 167 (10)	
**58**	14.96 ^ii^	Verbascoside/ isoverbascoside *O*-Pent-*O*-Caf_2_^L^ [46,51]	PEG	−35	1079	[1079]: 917 (76), 755 (93), 623 (100) [623]: 477 (7), 461 (100), 161 (17) [461]: 315 (100)	
**59**	15.48 ^ii^	Verbascoside/ isoverbascoside *O*-Pent-*O*-Caf_2_^L^ [46,51]	PEG	−35	1079	[1079]: 917 (63), 755 (82), 623 (100) [623]: 477 (11), 461 (100), 161 (14) [461]: 315 (100)	
**60**	15.59 ^ii^	Leucosceptoside A *O*-Pent-*O*-Caf_2_^L^ [46,51]	PEG	−35	1093	[1093]: 931 (64), 769 (83), 637 (100) [637]: 505 (18), 461 (100) [461]: 315 (100), 153 (14)	
**61**	16.47 ^ii^	Leucosceptoside A *O*-Pent-*O*-Caf_2_^L^ [46,51]	PEG	−35	1093	[1093]: 931 (60), 769 (85), 637 (100) [637]: 505 (15), 461 (100) [461]: 315 (100), 153 (11)	
**62**	17.97 ^ii^	Leonoside B *O*-Pent-*O*-Caf_2_^L^ [46,51]	PEG	−38	1239	[1239]: 1077 (22), 915 (94), 783 (100) [783]: 651 (4), 607 (100) [607]: 475 (100), 329 (25), 167 (12)	
**63**	18.87 ^ii^	Verbascoside/ isoverbascoside *O*-Pent-*O*-Caf_2_-*O*-Fer ^L^ [46,51]	PEG	−35	1255	[1255]: 1093 (52), 1079 (12), 931 (76), 755 (100) [755]: 623 (100) [623]: 477 (10), 461 (100), 161 (12) [461]: 315 (100)	
**64**	19.02 ^ii^	Verbascoside/ isoverbascoside *O*-Pent-*O*-Caf_2_-*O*-Fer ^L^ [46,51]	PEG	−35	1255	[1255]: 1093 (48), 1079 (9), 931 (78), 755 (100) [755]: 623 (100) [623]: 477 (11), 461 (100), 161 (4) [461]: 315 (100)	
**65**	11.21 ^iii^	1-*O*-Caffeoylquinic acid ^S^ [54]	CQA	−15	353	[353]: 191 (100), 179 (5)	399 ^FA^
**66**	11.48 ^iii^	4-*O*-Caffeoylquinic acid ^S^ [54]	CQA	−15	353	[353]: 191 (35), 179 (100), 135 (18)	399 ^FA^
**67**	12.32 ^iii^	Phaselic acid ^S^ [55]	CQA	−15	293	[293]: 179 (100)	339 ^FA^
**68**	12.98 ^iii^	5-*O*-Caffeoylquinic acid ^S^ [54]	CQA	−15	353	[353]: 191 (100), 179 (6), 135 (5)	399 ^FA^
**69**	14.17 ^iii^	3-*O*-Caffeoylquinic acid ^S^ [54]	CQA	−15	353	[353]: 191 (100), 179 (5), 135 (12)	399 ^FA^
**70**	14.36 ^iii^	6-Hydroxyluteolin *O*-HexA-*O*-Hex ^L^ [46,56,57]	HLG	−20	639	[639]: 477 (32), 301 (100)	
**71**	14.92 ^iii^	Luteolin *O*-HexA-*O*-Hex ^L^ [46,56]	LG	−20	623	[623]: 461 (28), 285 (100)	
**72**	15.29 ^iii^	Apigenin *O*-HexA-*O*-Hex ^L^ [46,58]	AG	−22	607	[607]: 445 (38), 269 (100)	
**73**	16.97 ^iii^	6-Hydroxyluteolin 7-*O*-GlcA ^S^ [46,56,57]	HLG	−20	477	[477]: 301 (100)	523 ^FA^
**74**	17.69 ^iii^	Luteolin 7-*O*-GlcA ^S^ [58]	LG	−20	461	[461]: 285 (100)	507 ^FA^
**75**	18.42 ^iii^	Luteolin *O*-*p*Cou-*O*-HexA-*O*-Hex ^L^ [46,59]	LGC	−25	769	[769]: 623 (100), 461 (25) [623]: 461 (39), 285 (100)	
**76**	18.91 ^iii^	Scutellarein 7-*O*-GlcA ^S^ [60]	SG	−30	461	[461]: 285 (100)	507 ^FA^
**77**	19.26 ^iii^	6-Hydroxyluteolin *O*-*p*Cou-*O*-HexA ^L^ [57,59]	LGC	−25	623	[623]: 477 (32), 301 (100)	
**78**	20.02 ^iii^	Apigenin 7-*O*-GlcA ^S^ [58]	AG	−20	445	[445]: 269 (100)	491 ^FA^
**79**	20.42 ^iii^	Luteolin *O*-HexA ^L^ [57,59]	LG	−20	461	[461]: 285 (100)	507 ^FA^
**80**	23.03 ^iii^	Luteolin *O*-*p*Cou-*O*-HexA ^L^ [54,56]	LGC	−25	607	[607]: 461 (15), 431 (2), 285 (100)	
**81**	24.85 ^iii^	Luteolin *O*-*p*Cou-*O*-HexA ^L^ [54,59]	LGC	−25	607	[607]: 461 (17), 431 (3), 285 (100)	653 ^FA^
**82**	27.91 ^iii^	Scutellarein *O*-*p*Cou-*O*-HexA ^L^ [59,60]	SGC	−30	607	[607]: 461 (35), 285 (100)	
**83**	29.12 ^iii^	Apigenin *O*-*p*Cou-*O*-HexA ^L^ [58]	AGC	−25	591	[591]: 445 (37), 415 (3), 269 (100)	637 ^FA^
**84**	29.93 ^iii^	Luteolin 7-*O*-(6’’-*O*-*p*Cou)-Glc ^S^ [58]	LGC	−20	593	[593]: 447 (27), 285 (100)	639 ^FA^
**85**	30.05 ^iii^	Apigenin *O*-*p*Cou-*O*-Hex ^L^ [58,59]	AGC	−30	591	[591]: 445 (53), 269 (100)	
**86**	30.94 ^iii^	Apigenin 7-*O*-(6’’-*O*-*p*Cou)-Glc ^S^ [58]	AGC	−30	591	[591]: 445 (42), 269 (100)	637 ^FA^
**87**	32.88 ^iii^	Luteolin *O*-*p*Cou_2_-*O*-HexA ^L^ [58,59]	LGC	−35	753	[753]: 607 (2), 461 (30), 285 (100)	
**88**	34.20 ^iii^	Apigenin *O*-*p*Cou_2_-*O*-HexA ^L^ [58,59]	AGC	−35	737	[737]: 591 (1), 445 (27), 269 (100)	
**89**	36.22 ^iii^	Apigenin *O*-*p*Cou_2_-*O*-Hex ^L^ [58,59]	AGC	−35	737	[737]: 591 (2), 445 (31), 269 (100)	
**90**	38.14 ^iii^	Apigenin *O*-*p*Cou_2_-*O*-Hex ^L^ [58,59]	AGC	−35	737	[737]: 591 (1), 445 (18), 269 (100)	

^a^ Chromatographic conditions: i—mode 1; ii—mode 2; iii—mode 3. ^b^ Compound identification was based on comparison of retention time, UV and MS spectral data with reference standard (S) or interpretation of UV and MS spectral data and comparison with literature data (L). ^c^ UV patterns as listed in Table S1: AG—apigenin glycoside; AGC—apigenin glycoside acylated with *p*-coumaric acid; CQA—caffeoylquinic acid; HLG—6-hydroxyluteolin glycoside; IG—iridoid glycoside; LG—luteolin glucoside; LGC—luteolin glycoside acylated with *p*-coumaric acid; PEG—phenylethanoid glycoside; SG—scutellarein glycoside; SGC—scutellarein glycoside acylated with *p*-coumaric acid. ^d^ CE—collision energy. ^e^ Mass spectrometric data: deprotonated ion [M–H]–, negative ionization/protonated ion [M+H]+, positive ionization. ^f^ Signal intensity (percentage). ^g^ Adduct ions: Na—with sodium [M+Na]+ in positive ionization; FA—with formic acid [(M-H)+HCOOH]- in negative ionization. Abbreviation used: Ac—acetyl; Caf—caffeoyl; pCou—*p*-coumaroyl; Fer—feruloyl; Glc—glucose; Hex—hexose; HexA—hexuronic acid; Pent—pentose.

To date, compounds with the above-mentioned chemical characteristics are still unknown. Reptoside and ajugoside as isomeric iridoid glycosides have mass spectrometric patterns close to their derivatives; however, it can be concluded that compounds **16** and **17** were the isomers of reptoside/ajugoside *O*-hexoside and compounds **31**–**33** were reptoside/ajugoside *O*-acetates with no known analogues in nature. Two iridoid glycosides, galiridoside (**4**) and antirrhinoside (**8**), were tentatively found in *G. bifida* extract using the literature data [47,49] as well as their *O*-hexosides, compounds **2** and **6,** and monoacetates, compounds **7**, **11**, and **12**.

As a result of the chromatographic separation, 30 iridoid glycosides were introduced as new components of the *G. bifida* metabolome, while three of the identified compounds (**3**, **4**, and **27**) were identified in previous works [7,8]. The majority of compounds found in *G. bifida* have either one, two, or three acetyl fragments bonded to the iridoid skeleton or/and carbohydrate moiety. The frequent presence of harpagide 8-*O*-acetate (**15**) [8,9], 6-desoxyharpagide 8-*O*-acetate (reptoside, **25**) [7], and ajugol 8-*O*-acetate (ajugoside, **27**) [7] among the *Galeopsis* species (*G. tetrahit*, *G. pubescens*, *G. ladanum*, *G. ladanum* subsp. *angustifolia*, *G. pyrenaica*, and *G. segetum*) indicates that acetylation is the distinctive pathway of iridoid glycoside derivatization in the genus (Table 1).

#### 2.1.2. Phenylethanoid Glycosides

Phenylethanoid glycosides were found in *G. bifida* for the first time, noticed mostly in the ethanol eluate of the SPE-polyamide cartridge. In total, 29 compounds (**34**–**43**, **45**–**49**, and **51**–**64**) were described, including seven phenylethanoid glycosides that were identified using reference standards, such as lavandulifolioside (**38**), verbascoside (**42**), leonoside A (**43**), isoverbascoside (**45**), leucosceptoside A (**46**), leonoside B (**47**), and martynoside (**52**) (Table 1). The main characteristic of phenylethanoid glycosides’ mass spectra is the loss of specific fragments of caffeic acid (−162 a.m.u.), ferulic acid (−176 a.m.u.), arabinose of pentose (−132 a.m.u.), rhamnose (−142 a.m.u.), and glucose or hexose (−162 a.m.u.). The ions with *m*/*z* 153 and 167 found in MS/MS spectra relate to the dehydrated fragment of dihydroxyphenylethanol and hydroxymethoxyphenylethanol, respectively, typical for the phenylethanoid glycosides of the Lamiaceae family [51,52]. On the basis of that feature, the remaining compounds were described as verbascoside/isoverbascoside derivatives with additional *O*-linked fragments of pentosyl-*O*-pentose (**34**–**36**), pentose (**40**) [51], caffeoyl-*O*-pentosyl-*O*-pentose (**48**, **49**, **51**), caffeoyl-*O*-pentose (**53**, **54**), di-*O*-caffeoyl-*O*-pentose (**58**, **59**), and feruloyl-di-*O*-caffeoyl-*O*-pentose (**63**, **64**) [46,51]. The same method was used for the identification of the leucosceptoside A (**39**, **41**,**55**, **56**, **60**, and **61**) and leonoside B derivatives (**57**, **62**). Despite the lack of scientific information regarding phenylethanoid glycosides in the *Galeopsis* genus, with the exception of *G. pubescens*, which contains martynoside and isomartynoside [16], lavandulifolioside, verbascoside, isoverbascoside, leonosides A and B, leucosceptoside A, and martynoside are frequent metabolites of Lamiaceous plants. For the tribe Stachydeae, which includes the *Galeopsis* genus, the phenylethanoid glycosides are taxonomic markers and have been found in *Ballota* [61], *Lamium* [62], *Phlomis* [63], *Stachys* [64], and *Sideritis* species [64]. Therefore, it was to be expected that verbascoside and its relatives would be found in *Galeopsis* species.

#### 2.1.3. Hydroxycinnamates

Caffeoylquinic acids (**65**, **66**, **68**, and **69**) and phaselic acid (**67**) were included in the hydroxycinnamates detected in the alkaline eluate of *G. bifida* extract. A comparison of retention times, UV-, and mass-spectra with reference standards allowed the identification of quinic acid derivatives, such as monocaffeoylated 1-*O*-(**65**), 3-*O*-(**69**), 4-*O*-(**66**), and 5-*O*-caffeoylquinic acids (**68**). To the best of our knowledge, none of the above-mentioned compounds have previously been identified in *G. bifida*.

#### 2.1.4. Neutral Flavone Glycosides

Two neutral flavone *O*-glycosides were found in *G. bifida*, specifically luteolin 7-*O*-glucoside (**44**) and apigenin 7-*O*-glucoside (**50**). Both are usual Lamiaceous flavonoids to detect early in *G. bifida* herb extract [17].

#### 2.1.5. Acidic Flavone Glycosides

Twenty flavone compounds (**70**–**90**) of acidic nature were found in the alkaline eluate of the SPE-polyamide cartridge. Two groups of flavonoids could be distinguished, specifically non-acylated and acylated flavone glycosides. In the first group, there were four known flavones including 6-hydroxyluteolin 7-*O*-glucuronide (**73**), luteolin 7-*O*-glucuronide (**74**), scutellarein 7-*O*-glucuronide (**76**), and apigenin 7-*O*-glucuronide (**78**), which were identified using reference standards. Compounds **74** and **78** were described previously in *G. bifida* [17]. The remaining non-acylated flavone glycosides demonstrated the sequential loss of fragments of hexose with *m*/*z* 162 and hexuronic acid with *m*/*z* 176, which is typical for the flavonoid hexosyl-hexuronides [65]. Under these circumstances, the possible structures of the compounds were 6-hydroxyluteolin 7-*O*-(X’’-*O*-glucosyl)-glucuronide (**70**), luteolin 7-*O*-(X’’-*O*-glucosyl)-glucuronide (**71**), and apigenin 7-*O*-(X’’-*O*-glucosyl)-glucuronide (**72**). The known analogs were found in *Sonchus* (luteolin 7-*O*-glucosylglucuronide) [66] and *Antirrhinum* plants (apigenin 7-*O*-glucosylglucuronide) [67]. Compound **79** gave a close mass spectrometric pattern to luteolin 7-*O*-glucuronide (**74**) while demonstrating a higher retention time, which is possible for its 3′ or 4′ isomers [68]. Luteolin 3′-*O*-glucuronide was found early in *Melissa offcinalis* [69].

Thirteen compounds were acylated flavone glycosides, two of which were identified after comparison with reference standards as luteolin 7-*O*-(6″-*p*-coumaroyl)-glucoside (**84**) and apigenin 7-*O*-(6″-*p*-coumaroyl)-glucoside (**86**), the latter of which has previously been mentioned as a component of *G. bifida* collected from Europe [17]. Compound **85** was isomeric to apigenin 7-*O*-(6″-*p*-coumaroyl)-glucoside (**86**) with the possible position of a *p*-coumaroyl group at the 2″-, 3″-, or 4″-C atom of glucose, as found in *Echinops echinatus* [70,71] and *Sideritis raeseri* [72]. The two flavones with the highest chromatographic mobility (compounds **89** and **90**) were di-*O*-*p*-coumaroyl-hexosides of apigenin, with possible known structures of apigenin 7-*O*-(2″,6″-di-*O*-*p*-coumaroyl)-glucoside and apigenin 7-*O*-(4″,6″-di-*O*-*p*-coumaroyl)-glucoside from *Anisomeles ovata* [73], or apigenin 7-*O*-(3″,6″-di-*O*-*p*-coumaroyl)-glucoside from *Stachys lanata* [74]. Five flavones presented the gradual loss of *p*-coumaroyl (−146 a.m.u.) and hexuronyl fragments (−176 a.m.u.) in the mass spectra data, indicating the presence of *p*-coumaroyl-hexuronyl structures of sugar linked with 6-hydroxuluteolin (**77**), luteolin (**80**,**81**), scutellarein (**82**), and apigenin (**83**). Compound **75** was a luteolin glucoside, which includes *p*-coumaroyl, hexosyl, and hexuronyl fragments, while compounds **87** and **88** were di-*O*-*p*-coumaroyl-hexuronides of luteolin and apigenin, respectively. Coumaroylated analogs of hexuronides of apigenin, scutellarein, luteolin, and 6-hydroxyluteolin are still unknown.

#### 2.1.6. Chemotaxonomic Significance of *G. bifida* Metabolites

As a result of the chromatographic research of *G. bifida*, approximaetly 100 metabolites of various chemical groups were identified. When choosing compounds that may have chemotaxonomic significance, special attention was paid to the iridoid glycosides, phenylethanoid glycosides, phenylpropanoids, and flavone glycosides.

Earlier attempts have been made to use iridoid glycosides as marker compounds within the genus *Galeopsis* and the Lamiaceae family [7,8,9]. The use of individual iridoids for the chemical division of the genus *Galeopsis* into the subgenera *Ladanum* and *Galeopsis* (*Tetrahit*) was unsuccessful. The assumption that harpagide 8-*O*-acetate, reptoside, and ajugoside are characteristic only of the species of subgenus *Ladanum* [7] is not supported by our data. In view of the fact that harpagide and its 8-*O*-acetate are more widespread in the Lamiaceae family, especially in tribe Stachydeae in genus *Betonica* and *Stachys* [44], the known conclusions about the applicability of specific iridoid glycosides for taxonomic purposes should be revised.

Phenylethanoid glycosides are widespread in the Lamiaceae family, especially in genera closely related to *Galeopsis*, such as *Ballota* [61], *Lamium* [62], *Phlomis* [63], and *Stachys* [64]. A high occurrence of verbascoside and isoverbascoside is characteristic of many species of Lamiaceae [75], especially the species belonging to the subfamily Stachyoideae [76]. The same principles apply to chlorogenic acids, which are found in most species of the Lamiaceae genus [76]. These features of the chemical composition of *G. bifida*, as well as the genus *Galeopsis*, indicate a low value of phenylethanoid glycosides and phenylpropanoids in terms of chemotaxonomic purposes.

Currently, information exists in the literature connecting flavone glycosides to nine species of the genus *Galeopsis* (Table 1). Summarizing the known data and the results of this study, it can be noted that flavones of the *Galeopsis* (*Tetrahit*) subgenus contain compounds with the 5,7- and/or 5,6,7-type of substitution, such as apigenin, scutellarin, luteolin, and chrysoeriol [9,17]. The main types of glycosides are the 7-*O*-glucosides and 7-*O*-glucuronides. Despite the fact that a detailed study of acylated flavone glycosides was carried out only for *G. bifida* (in the present study), similarities between chemical characteristics within the subgenus *Galeopsis* (*Tetrahit*) suggest the presence of these compounds in other species of the subgenus. Flavonoids of the Ladanum subgenus include flavones with a 5,7,8-trisubstituted ring A, such as isoscutellarin (5,7,8,4′-tetrahydroxyflavone), hypoletin (5,7,8,3′, 4′-pentahydroxyflavone), 8-hydroxychrysoeriol (5,7,8,4′-tetrahydroxy-3’-methoxyflavone), and 8-hydroxydiosmethin (5,7,8,3′-tetrahydroxy-4′-methoxyflavone) [17]. The carbohydrate moiety of these glycosides is a rare disaccharide 2-*O*-allosyl-glucose, which may have one or two acetyl residues at the C-6 position of allose and/or glucose.

It should be noted that there is a clear differentiation between the chemical characteristics of the species belonging to different subgenera: the presence of 5,7,8-trihydroxyflavones has not been established in species of the subgenus *Galeopsis* (*Tetrahit*) [17]; this finding is congruent with our research on *G. bifida*. In previous studies, two flavones have been isolated from *G. ladanum*, specifically ladanein (5,6-dihydroxy-7,4′-dimethoxyflavone) and ladanetin (5,6,4′-trihydroxy-7-methoxyflavone), the structural type of which does not correspond to the typical structure for the subgenus *Ladanum* [18]. Tomas-Barberan et al., following a chromatographic study of the genus *Galeopsis*, demonstrated that flavones with a hydroxy group at the C-6 position are not characteristic of the subgenus *Ladanum* [77]. Following this report [18], the presence of ladanein in the Lamiaceae family was also revealed in the tribes Ocimeae (*Ocimum* [78], *Orthosiphon* [79], *Lavandula* [80], *Plectranthus* [81]), Marrubieae (*Ballota* [82], and *Marrubium* [83]) and Nepetoideae (*Rosmarinus* [84], *Salvia* [85], and *Thymus* [77]), however, never in the tribe *Galeopsis*. A similar situation was observed with the flavone ladanetin, which is characteristic of the genus *Dracocephalum* [86], but not of *Galeopsis*. Considering the above, the presence of ladanein and ladanetin in *G. ladanum* and the genus *Galeopsis* remains doubtful.

### 2.2. Quantification of G. Bibida Metabolites: Organ Distribution and Two Siberian Chemotypes

#### 2.2.1. Distribution of Selected Metabolites in *G. bifida* Organs

Studying the distribution of metabolites in the organs of medicinal plants is important for correctly selecting the part of the plant that contains the highest concentration of bioactive compounds. Due to the fact that *G. bifida* is harvested as a full-plant (including roots), it became necessary to determine the levels of individual compounds in the different parts of the plant. To do that, we used a quantitative HPLC-MS assay, which made it possible to determine the content of 18 compounds in the leaves, flowers, stems, and roots of *G. bifida* (Table 3). For quantification, we chose two iridoid glycosides (harpagide and harpagide 8-*O*-acetate), six phenylethanoid glycosides (verbascoside, isoverbascoside, lavandulifolioside, leucosceptoside A, and leonosides A and B), four caffeoylquinic acids (1-*O*-, 3-*O*-, 4-*O*-, and 5-*O*-caffeoylquinic acid), and six flavone glycosides (7-*O*-glucuronides of luteolin, apigenin, 6-hydroxyluteolin and scutellarein and 7-*O*-(6″-O-*p*-coumaroyl)-glucosides of luteolin, and apigenin).

The quantification data demonstrated an uneven distribution of the compounds within the various organs of *G. bifida*. Two dominant iridoid glycosides, harpagide 8-*O*-acetate and harpagide, were found in higher levels in the leaves (25.69 and 11.35 mg/g dry plant weight) and stems (18.53 and 9.37 mg/g dry plant weight), in contrast to the flowers and roots, which contained total iridoid glycosides content levels of 15.55 and 2.12 mg/g, respectively. The highest total phenylethanoid glycoside content was found in the leaves (61.38 mg/g) of *G. bifida*, followed by the flowers (50.06 mg/g), stems (18.47 mg/g), and roots (7.32 mg/g). The main compound was verbascoside, which amounted to 21.56, 18.98, 5.32, and 2.63 mg/g of the dry weight of the leaves, flowers, stems, and roots, respectively. Above all, attention should be drawn to the high levels of isoverbascoside (2.08–14.88 mg/g) and lavandulifolioside (1.57–16.37 mg/g) in the organs of *G. bifida*.

Caffeoylquinic acid showed higher concentrations in the leaves (47.52 mg/g in total), dominated by 5-*O*-caffeoylquinic acid (45.20 mg/g). Meanwhile, the total level of caffeoylquinic acid in the other organs of *G. bifida* was 16.01 mg/g in the flowers, 9.21 mg/g in the stems, and 2.90 mg/g in the roots. Flavone glycosides comprised a large group of compounds with high content in the leaves (80.56 mg/g in total) and flowers (53.48 mg/g in total), with the predominant luteolin 7-*O*-glucuronide contributing 29.73 and 39.63 mg/g of the leaf and flower dry weights, respectively. Apigenin 7-*O*-glucuronide was the second highest-level flavonoid in the leaves (19.32 mg/g) and scutellarein 7-*O*-glucuronide was also in high levels in the flowers (5.22 mg/g). The content of non-acylated flavone glycosides was greater than acylated derivatives in all organs of *G. bifida*. These results make obvious the fact that the roots are poor in metabolites and, thus, the above-ground parts of *G. bifida* (leaves, flowers, and stems) should be recommended for use in medical applications.

Due to the absence of information regarding the metabolite content of *Galeopsis* species, we compared the results of our study with known data concerning the metabolite content of other Lamiaceous species. Háznagy-Radnai et al. studied the total content of iridoid glycosides in ten *Stachys* species collected in Bulgaria, and found that the levels reached 15.2 mg/g in *S. officinalis* leaves, 16.8 mg/g in *S. officinalis* flowers, and 14.7 mg/g in *S. recta* roots [87]. The level of harpagide and harpagide 8-*O*-acetate in *Leonurus* species from central regions of Russia were found to be 0.11–0.37 and 0.10–0.37 mg/g in *Leonurus quinquelobatus* herb, respectively, and 0.06 and 0.04 mg/g in *Leonurus cardiaca* herb, respectively [88]. Despite iridoid accumulation, some *Leonurus* species have been characterized by high phenylethanoid glycoside content, varying from 3.09 mg/g in *L. quinquelobatus* to 26.17 mg/g in *L. cardiaca*, and high verbascoside levels in six Siberian *Leonurus* species, ranging from 0.89–3.66 mg/g [89]. Flavonoids, as the most studied group of Lamiaceae phenolics, were at highest levels in the family and selected species. For example, the levels of flavonoids in herbs of *Stachys byzantine*—11.1 mg/g, *Salvia officinalis*—5.12 mg/g, *Mentha suaveolens*—3.9 mg/g [90], *Mentha piperita*—30.2–63.2 mg/g [91], *Panzerina lanata*—29.3 mg/g [92], *Thymus baicalensis*—18.4 mg/g, *Thymus sibiricus*—26.5 mg/g [93], *Nepeta glutinosa*—7.3–10.2 mg/g, *Ziziphora pamiroalaica*—8.3–10.1 mg/g [94], and *Dracocephalum palmatum*—10.5–35.4 mg/g [95] are known. Undoubtedly, the herb of *G. bifida* is a good source of iridoids and phenolic compounds that contain a comparable or greater level of phytocomponents.

#### 2.2.2. Two Siberian Chemotypes of *G. bifida*

**Figure 2:**
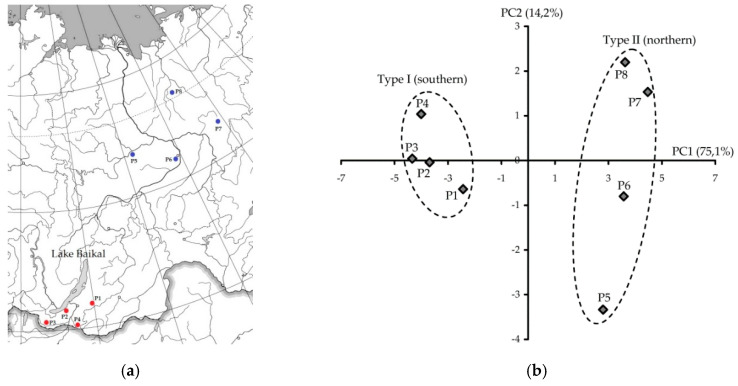
(**a**) Location of *Galeopsis bifida* populations. (**b**) Results of principal component analysis (PCA) used the content of eighteen compounds in *G. bifida* leaves from eight Siberian populations. P1–P8—number of *G. bifida* population.

Investigations into geographical variations of the chemical profiles of plants allowed us to understand the level of stability of metabolic pathways of selected species, as well as the power of climatic influence on the plant populations. This is particularly relevant for species with a wide area of distribution, such as the common hemp–nettle, which is located across the whole of Eurasia. In this study, we analyzed eight Siberian populations of *G. bifida* located at the southern (Buryatia Republic, populations P1–P4) and northern (Sakha Yakutia Republic, populations P5–P8) borders of the Russian area (Figure 2a).

Moreover, it should be pointed out that we used the leaf samples only because they contained the highest content of metabolites. The results of HPLC-PAD-ESI-tQ-MS profiling of *G. bifida* extracts demonstrated the stability of qualitative metabolite patterns in all samples analyzed; however, the quantification data indicated various levels of the selected compounds (Table 4). Considering the features of metabolite accumulation in *G. bifida* leaves, the following conclusions can be drawn:

The specificity of iridoid glycoside accumulation in the southern populations of *G. bifida* lies in the fact that southern and northern populations have similar content levels of harpagide and its *O*-acetate (8.57–18.33 mg/g vs. 9.35–14.53 mg/g, respectively), while northern populations have a much greater content of harpagide 8-*O*-acetate in contrast to southern populations (<0.10–4.14 mg/g vs. 24.52–31.82 mg/g, respectively).

The level of phenylethanoid glycosides in northern populations (61.16–69.79 mg/g) was higher than in southern populations (6.21–12.73 mg/g), while the selected compounds of isoverbascoside and leonoside B were trace components in southern populations, in contrast to northern populations (12.76–18.67 and 1.57–1.93 mg/g, respectively).

Caffeoylquinic acids were detected in all populations, however, their concentrations varied from 35.02 to 44.90 mg/g and from 5.33 to 13.83 mg/g in samples from Sakha Yakutia Republic and Buryatia Republic, respectively.

Total flavone glycoside content in *G. bifida* leaves were similar from southern and northern populations (62.26–82.37 mg/g vs. 70.99–93.65 mg/g, repsectively); however, differing accumulations of non-acylated and acylated flavonoids were observed. Non-acylated flavone glucuronides were the predominant species in southern populations (61.74–82.26 mg/g) accounting for 99.2–100% of the total flavonoid content. Meanwhile, in the plants collected from northern populations, the content of non-acylated and acylated flavonoids was 39.7–66.5% and 33.5–60.3% of the total flavonoid content, respectively, and in the most northern samples (P7, P8), the content of the rare 7-*O*-(6″-*O*-*p*-coumaroyl)-glucosides of luteolin and apigenin were 37.99–56.46 mg/g.

The results of the principal component analysis (PCA) confirmed the division of the studied *G. bifida* population into two types: type I or southern type, located on the left side of the diagram, and type II or northern type, located on the right side of the diagram (Figure 2b). The total scores plot of PCA in a two-component model amounted to 89.3% of the total variability. These results indicate the existence of at least two chemotypes of G. bifida in the Siberian region: the southern chemotype, with the predominance of non-acylated flavone glucuronides, and the northern chemotype, with a high content of acetylated iridoids, phenylethanoid glycosides, caffeoylquinic acids, and acylated flavone glycosides. Obviously, additional studies need to be performed to ensure the correctness of this theory.

In the debate regarding possible reasons for chemical variation between the southern and northern populations of *G. bifida*, climatic differences between the Buryatia Republic, with a warm humid continental climate, and the Sakha Yakutia Republic, located in the subarctic area of Siberia, should be mentioned. The cold climate of the northern territories promotes the accumulation of compounds such as acetylated iridoids, phenylethanoid glycosides, caffeoylquinic acids, and acylated flavone glycosides in *G. bifida* plants, while warm climates result in high concentrations of the non-acetylated iridoid glycosides and non-acylated flavone glucuronides. This early comparative data regarding the chemical composition of plants collected from the various regions of Siberia demonstrate greater storage ability in the northern plant populations for flavonoids [53,56,96], simple phenolics [97], ellagitannins [98], iridoids [99], caffeoylquinic acids [100], sesquiterpenes [101], and coumarins [102].

### 2.3. Bioactivity of G. bifida Extracts: Acute Toxicity and Antioxidant Potential

Existing data concerning the possible toxicity of *Galeopsis* extracts [15,16] inspired us to determine the acute toxicity of *G. bifida* methanol extracts (GBME) prior to proceeding with other pharmacological experiments. Intraperitoneal administration of GBME from the southern population, P3, and northern population, P7, in doses of 1–3000 mg/kg, did not cause the death of experimental animals (mice) during a week. According to our data, this shows that GBME is a plant extract with low toxicity.

Owing to the high content of the phenolic compounds in *G. bifida* plant material and dry extracts (Table S2), especially compounds with expressed antioxidant potential, such as phenylethanoid glucosides [50], caffeoylquinic acids [100], and flavone glycosides [95], we studied the antioxidative properties of GBME from eight Siberian populations. Six assays were chosen for investigation, including cavenging capacity against free radicals of 2,2-diphenyl-1-picrylhydrazyl radical (DPPH), 2,2′-azino-bis(3-ethylbenzothiazoline-6-sulfonic acid) cation radical (ABTS), and superoxide radical, as well as ferric reducing antioxidant power, oxygen radical absorbance capacity, and carotene bleaching assay. Trolox was used as a reference substance (Table 5).

All studied GBMEs demonstrated high effectiveness as antioxidants in the six assays, with potential ranging from 286.6–632.4 μM Trolox-eq./g in scavenging of DPPH radicals, 293.4–693.0 μM Trolox-eq./g in scavenging of ABTS radicals, 182.4–363.7 μM Trolox-eq./g in scavenging of superoxide radicals, 103.9–361.2 μM Trolox-eq./g in ferric reducing antioxidant power, 253.0–631.0 μM Trolox-eq./g in oxygen radical absorbance capacity, and 298.3–734.8 μM Trolox-eq./g in carotene bleaching assay.

The power of GBME prepared from the southern populations, P1–P4, was lower than the power of GBME prepared from the northern populations, P5–P8. This phenomenon is obviously caused by the higher content of phenolics in the extracts from P5–P8. The data regression analysis of “antioxidant activity–compound content” relationships confirmed these findings via high values of the regression coefficients (*r*^2^) of the linear equations (>0.5) (Table 6).

These conclusions are reinforced by the known pharmacological data relating to *Galeopsis* plants reporting the low toxicity of *G. ladanum* extract [29] and the good antioxidant potential of *G. speciosa* extract in the DPPH assay (IC_50_ 2.85–4.00 μg/mL), phosphomolybdenum assay, and linoleic acid peroxidation assay (64.5%) [30]. It can be said for Lamiaceous plants as a whole that their extracts are safe and effective antioxidants, such as those that have traditionally been used, such as skullcaps [65], motherworts, sages, dead-nettles [30], lemon balm, peppermints [103], and thymes [104]. Hemp nettle is a good addition to the list of known medicinal plants with potential as bioactive.

## 3. Materials and Methods

### 3.1. Plant Material and Chemicals

Samples of *Galeopsis bifida* were collected in the eight Siberian regions in the flowering stage on the same day (20.VI.2019) (Table 7). The species was authenticated by Dr. N.I. Kashchenko (IGEB SB RAS, Ulan-Ude, Russia) and Professor N.K. Chirikova (North-Eastern Federal University, Yakutsk, Russia). The plant material was dried in the ventilated heat oven at 40 °C within 3–4 days and stored at 3–4 °C before analysis. The reference compounds were purchased from ChemFaces (Wuhan, Hubei, PRC), Extrasynthese (Lyon, France), MedKoo Biosciences Inc. (Morrisville, NC, USA), Sigma-Aldrich (St. Louis, MO, USA), Toronto Research Chemicals (North York, ON, Canada), VILAR Corp. (Moscow, Russia), or isolated early from the various plants in our laboratory [55,57,100,105,106] (Table S3). Selected chemicals were from Sigma-Aldrich—acetonitrile for HPLC (Cat. No 34851, ≥99.9%); 2,2′-azino-bis(3-ethylbenzothiazoline-6-sulfonic acid) diammonium salt (Cat. No A1888, ≥98%); 2,2′-azobis(2-methylpropionamidine) dihydrochloride (Cat. No 440914, ≥97%); β-carotene (Ct. No C4582, ≥95%); 2,2-diphenyl-1-picrylhydrazyl radical (Cat. No 281689, ≥97%); formic acid (Cat. No 33015, ≥98%); methanol (Cat. No. 322415, ≥99.8%); myoglobin (Cat. No M0630, ≥95%); pyrogallol (Cat. No P0381, ≥98%); 2,4,6-tri(2-pyridyl)-1,3,5-triazine (Cat. No 93285, ≥99%); and Trolox (Cat. No 238813, ≥97%).

### 3.2. Plant Extracts Preparation

The extract of *G. bifida* herb for the qualitative analysis was prepared from the total aerial part (leaves, flowers, and stems) of the P5 sample and 100 g of the dry powdered herb was extracted by 60% ethanol with sonication (60 min, 50 °C, ultrasound power 100 W, frequency 35 kHz) for that purpose. Liquide extract was filtered through the filter paper and concentrated in vacuo until dryness. The yield of the dry extract from *G. bifida* herb was 19% from dry plant weight. The dry extracts of leaves from the samples P1–P8 for the quantitative analysis and study of biological activity were produced using the same technology with the yields 28% (P1), 24% (P2), 25% (P3), 29% (P4), 33% (P5), 35% (P6), 31% (P7), and 30% (P8) of dry plant weight.

### 3.3. Polyamide Solid-Phase Extraction

The separation of the extract of *G. bifida* herb before qualitative chromatographic analysis was realized with solid-phase extraction (SPE) on the polyamide cartridges Chromabond (Polyamide 6; 6 mL, 1000 mg; Sorbent Technologies, Inc., Norcross, GA, USA) preconditioned with methanol (50 mL) and water (70 mL). The dry extract (100 mg) was dissolved in 25% methanol (10 mL), centrifuged (6000× *g*, 15 min), and the supernatant volume reached 10 mL in the volumetric flask (10 mL; solution A). An aliquote of solution A (5 mL) was mixed with 100 μL of trifloroside solution (internal standard-1; 2 mg/mL in 20% methanol), 100 μL of scopoletin 7-*O*-neohespridoside solution (internal standard-2; 2.5 mg/mL in 40% methanol), and 50 μL of 3,5-di-*O*-feruloylquinic acid solution (internal standard-3; 1 mg/mL in 40% methanol), and the mixture was passed through preconditioned polyamide SPE-cartridge eluted with water (40 mL; eluate I), 85% methanol (50 mL; eluate II), and 0.45% NH_3_ in methanol (50 mL; eluate III). Eluates I, II, and II were concentrated in vacuo, dissolved in 1 mL of methanol, and stored at 4 °C before chromatographic analysis (Section 3.4).

### 3.4. High-Performance Liquid Chromatography with Photodiode Array Detection and Electrospray Ionization Triple Quadrupole Mass Spectrometric Detection (HPLC-PDA-ESI-tQ-MS)

Qualitative chromatographic analysis of metabolic profiles of *G. bifida* extracts was done by high-performance liquid chromatography with photodiode array detection and electrospray ionization triple quadrupole mass spectrometric detection (HPLC-PDA-ESI-tQ-MS) technique using a liquid chromatograph LC-20 Prominence coupled with photodiode array detector SPD-M30A (wavelength range 200–600 nm), and triple-quadrupole mass spectrometer LCMS 8050 (all Shimadzu, Columbia, MD, USA) and C18 columns. Two-eluent gradient elution was used for successful separation of compounds in three chromatographic modes: mode 1 (separation of SPE-polyamide eluate I)—column ProteCol™ C18 HPH125 (250 × 4.6 mm, Ø 5 μm; Trajan Scientific Australia Pty Ltd., Ringwood, Victoria, Australia); column temperature 25 °C; eluents A, 0.2% HCOOH in water; eluent B, MeCN; gradient program: 0–2 min 5–6% B, 2–9 min 6–11% B, 9–15 min 11–25% B, 15–20 min 25–55% B, 20–25 min 55–5% B; mode 2 (separation of SPE-polyamide eluate II)—column GLC Mastro (150 × 2.1 mm, Ø 3 μm; Shimadzu, Kyoto, Japan); column temperature 30 °C; eluents A, 0.5% HCOOH in water; eluent B, 0.5% HCOOH in MeCN; gradient program: 0–2 min 5–6% B, 2–9 min 6–11% B, 9–15 min 11–25% B, 15–20 min 25–55% B, 20–25 min 55–5% B; mode 3 (separation of SPE-polyamide eluate III and quantitative analysis of *G. bifida* organs and extracts)—column GLC Mastro (150 × 2.1 mm, Ø 3 μm; Shimadzu, Kyoto, Japan); column temperature 30 °C; eluents A, 0.5% HCOOH in water; eluent B, 0.5% HCOOH in MeCN; gradient program: 0–5 min 5–10% B, 5–10 min 10–15% B, 10–22 min 15–20% B, 22–28 min 20–34% B, 28–35 min 34–52% B, 35–40 min 52–80% B, 40–50 min 80–5% B. The injection volume was 1 μL and the elution flow 100 μL/min. The UV-Vis spectra were registered in the spectral range of 200–600 nm. Mass spectrometric detection was performed both in negative and positive ESI mode and the temperature levels of ESI interface, desolvation line, and heat block were 300 °C, 250 °C, and 400 °C, respectively, and the flow of nebulizing gas (N_2_), heating gas (air), and collision-induced dissociation gas (Ar) were 3 L/min, 10 L/min, and 0.3 mL/min, respectively. The mass spectra were registered as 3 kV source voltage and collision energy +15–+25 eV in the positive mode and −15–35 eV in the negative mode by the scanning range of *m*/*z* 50–2000. LabSolution’s workstation software with the inner LC-MS library was used to managing the LC-MS system. The final identification of metabolites was done after an integrated analysis of retention time, ultraviolet, and mass spectra with the reference samples and/or literature data.

### 3.5. Metabolite Quantification

Quantification of compounds in *G. bifida* extracts was realized in chromatographic conditions (mode III), as described above (Section 3.4) and HPLC-MS data (full scan MS, peak area) were used for calculation. Eighteen metabolites were quantified and seventeen solutions [harpagide, harpagide 8-*O*-acetate, verbascoside, isoverbascoside, leucosceptoside A, leonosides A and B, 1-*O*-, 3-*O*-, 4-*O*-, 5-*O*-caffeoylquinic acid, luteolin 7-*O*-glucuronide, apigenin 7-*O*-glucuronide, 6-hydroxyluteolin 7-*O*-glucuronide, scutellarein 7-*O*-glucuronide, luteolin 7-*O*-(6″-O-*p*-coumaroyl)-glucoside, and apigenin 7-*O*-(6″-O-*p*-coumaroyl)-glucoside] were prepared after careful weighing (10 mg) and dissolution in the methanol-DMSO mixture (1:1) in volumetric flasks (10 mL). Lavandulifolioside content was expressed as verbascoside equivalents. To build reference standard calibration curves, the stock solutions were diluted with methanol (1–100 µg/mL), chromatographed, and MS peak area data were used to plot “concentration–peak area” graphs. The validation criteria (correlation coefficients, *r*^2^; standard deviation, *S*_YX_; limits of detection, LOD; limits of quantification, LOQ; and linear ranges) were calculated as described previously [107] (Table S4). All analyses were carried out five times, and the data were expressed as mean value ± standard deviation (S.D.). For the analysis of *G. bifida* plant samples (leaves, flowers, stems, and roots), pulverized material (200 mg) was extracted with 60% ethanol (5 mL) twice by sonication (20 min, 50 °C, ultrasound power 100 W, frequency 35 kHz), followed by centrifugation (6000× *g*, 20 min) and filtering (0.22-μm PTFE syringe filter) to the volumetric flask (10 mL). The samples of *G. bifida* extracts were prepared the same way using 50 mg of dry material.

### 3.6. Acute Toxicity

Experiments were performed on adult male C57BL/6 mice (body weight range 80–100 g; 6–8 weeks of age) obtained from the ‘Pushchino’ Laboratory Animal Breeding House (Moscow, Russia). Animals were housed at 22 °C under a 12/12 light/dark cycle, with free access to food and water. Acute toxicity experiments (LD_50_) was determined using recommendations of the Guidelines for Preclinical Drug Trials [108] after oral administration of *G. bifida* extracts (samples P3 and P7) by gavage at the doses of 1 (8 animals), 10 (8 animals), 100 (10 animals), 1000 (10 animals), and 3000 (10 animals) mg/kg in a volume 10 mL/kg. The animals were continually observed for a week and there were no clinical signs of toxicity or mortality in the experimental groups. The experimental procedure was authorized by the Institute of General and Experimental Biology’s Ethical Committee (protocol No LM-0324, 27.01.2012) before starting the study and was conducted under the internationally accepted principles for laboratory animal use and care.

### 3.7. Antioxidant Activity

Microplate spectrophotometric assays were used to study the scavenging activity of *G. bifida* extracts against the 2,2-diphenyl-1-picrylhydrazyl radical and the 2,2′-azino-bis(3-ethylbenzothiazoline-6-sulfonic acid) cation radical, as described earlier [53,95] and the superoxide radicals scavenging capacity was determined using pyrogallol auto-oxidation assay [109]. Ferric reducing antioxidant power was determined by spectrophotometrical assay and used the reduction of the Fe^3+^-2,4,6-tri(2-pyridyl)-1,3,5-triazine complex to the Fe^2+^ at low pH [110]. The fluorimetric method of peroxyl radical generation by thermal decomposition of 2,2′-azobis(2-amidino-propane) dihydrochloride was used to measure the oxygen radical absorbance capacity assay [111] and peroxide-radical-induced destruction of the β-carotene was used in the spectrophotometric carotene-bleaching assay [112]. Trolox, as a reference standard (1–100 μg/mL in methanol), was used for the expression of the values of antioxidant parameters as μmol Trolox-equivalents/g of dry weight. All the analyses were carried out five times and the data were expressed as mean value ± standard deviation (SD).

### 3.8. Statistical and Multivariate Analysis

Statistical analyses were performed by one-way analysis of variance, and the significance of the mean difference was determined by Duncan’s multiple range test. Differences at *p* < 0.05 were considered statistically significant. The results are presented as mean values ± standard deviations (S.D.) of some replicates. The linear regression analysis and generation of calibration graphs were conducted using Advanced Grapher 2.2 (Alentum Software Inc., Ramat-Gan, Israel). Principal component analysis based on a data matrix (18 markers × 8 samples) was performed using Graphs 2.0 utility for Microsoft Excel (Komi NTc URO RAN, Syktyvkar, Russia) to generate an overview for group clustering.

## 4. Conclusions

*Galeopsis bifida* is a ruderal synanthropic species found throughout most of Eurasia. Early ethnopharmacological information has not been scientifically confirmed in the modern world; therefore, the use of this species is not widespread. In the course of this study, it was shown that *G. bifida* is characterized by the ability to accumulate phenolic compounds of different classes. In particular, the composition of *G. bifida* phenylpropanoids was established for the first time and it was shown that these compounds are represented by caffeoylquinic acids, as well as phenylethanoid glycosides. Flavonoids of this plant species consist of flavones in the form of *p*-coumaroyl glucosides and glucuronides. Of the 90 identified compounds, 82 were found in *G. bifida* for the first time. The finding of organ specificity among the accumulation of phenolic compounds in *G. bifida* indicates a greater practical significance of the aerial part of this species due to the ability of leaves and flowers to accumulate individual compounds. In the course of the study of the Siberian populations of *G. bifida*, the existence of two chemotypes characterized by geographical confinement was shown. This phenomenon can be important when choosing locations to collect plant materials from regarding specific parameters of their chemical composition. For the first time, a study of the pharmacological properties of *G. bifida* was carried out and it was found that its extracts can be considered as low-toxic antioxidant agents.

Considering the early ethnopharmacological information on the use of *G. bifida*, as well as data on its chemical composition, it can be assumed that recommendations for use of this species in the treatment of liver and stomach diseases, as well as many other illnesses, are due to its high content of compounds with antioxidant and anti-inflammatory activity, such as verbascoside, 3-*O*-caffeoylquinic acid, luteolin, and apigenin glycosides. In this regard, we conclude that the synanthropic plant species *G. bifida* is not just a weedy and unimportant plant, but instead, has great potential as a medicinal species and, thus, research into this species should be continued.

## Figures and Tables

**Table 1:** Known compounds of *Galeopsis* genus (literature data).

Compound	Found in *Galeopsis* Species of Subgenus	
	*Galeopsis (Tetrahit)*	*Ladanum*
Iridoids		
6-Desoxyharpagide	*G. pubescens* [6]	
	*G. tetrahit* [7]	
Harpagide	*G. bifida* [8]	*G. ladanum* [9]
	*G. pubescens* [9]	*G. ladanum* subsp. *angustifolia* [9]
	*G. speciosa* [9]	*G. pyrenaica* [8]
	*G. tetrahit* [9]	*G. reuteri* [8]
		*G. segetum* [9]
Harpagide 8-*O*-acetate	*G. pubescens* [8]	*G. ladanum* [8]
		*G. ladanum* subsp. *angustifolia* [9]
		*G. pyrenaica* [8]
		*G. segetum* [8]
Galiridoside	*G. bifida* [8]	*G. ladanum* [8]
	*G. pubescens* [8]	*G. pyrenaica* [8]
	*G. speciosa* [9]	*G. segetum* [8]
	*G. tetrahit* [10]	
Gluroside	*G. pubescens* [6]	
	*G. tetrahit* [6]	
Reptoside	*G. pubescens* [7]	*G. ladanum* [7]
	*G. tetrahit* [7]	*G. ladanum* subsp. *angustifolia* [7]
		*G. pyrenaica* [7]
		*G. segetum* [7]
Ajugoside	*G. bifida* [7]	
	*G. tetrahit* [7]	
Antirrhinoside		*G. segetum* [7]
Antirrhinoside 5-*O*-glucoside		*G. segetum* [7]
Daunoside	*G. pubescens* [7]	
	*G. tetrahit* [7]	
8-Epiloganin		*G. ladanum* subsp. *angustifolia* [11]
Diterpenoids		
Hispanolone		*G. ladanum* subsp. *angustifolia* [12]
Galeopsin		*G. ladanum* subsp. *angustifolia* [12]
Pregaleopsin		*G. ladanum* subsp. *angustifolia* [12]
Galepsitrione		*G. ladanum* subsp. *angustifolia* [13]
Galeolone		*G. ladanum* subsp. *angustifolia* [13]
Galepsinolone		*G. ladanum* subsp. *angustifolia* [13]
Hispanone		*G. ladanum* subsp. *angustifolia* [13]
Galeuterone		*G. reuteri* [14]
Pregaleuterone		*G. reuteri* [14]
Triterpenoids		
Hederagenin		*G. ladanum* subsp. *angustifolia* [13]
Benzoic acids		
4-Hydroxybenzoic acid		*G. segetum* [15]
Vanillic acid		*G. segetum* [15]
Hydroxycinnamates		
*p*-Coumaric acid		*G. segetum* [15]
Caffeic acid		*G. segetum* [15]
Ferulic acid		*G. segetum* [15]
Martynoside	*G. pubescens* [16]	
Isomartynoside	*G. pubescens* [16]	
Flavones		
5,7,4′-Trisubstituted flavones		
Apigenin		*G. segetum* [15]
Apigenin 7-*O*-glucoside	*G. bifida* [17]	*G. ladanum* [17]
	*G. pubescens* [17]	*G. ladanum* subsp. *angustifolia* [17]
	*G. speciosa* [9]	*G. pyrenaica* [17]
	*G. tetrahit* [17]	*G. × wirtgenii* [17]
Apigenin 7-*O*-(6″-*O*-*p*-coumaroyl)-glucoside	*G. bifida* [17]	*G. pyrenaica* [17]
	*G. pubescens* [17]	*G. segetum* [17]
	*G. speciosa* [9]	*G. × wirtgenii* [17]
	*G. tetrahit* [17]	
Apigenin 7-*O*-glucuronide	*G. bifida* [17]	*G. ladanum* [17]
	*G. pubescens* [17]	*G. ladanum* subsp. *angustifolia* [17]
	*G. speciosa* [9]	*G. pyrenaica* [17]
	*G. tetrahit* [17]	*G. segetum* [17]
		*G. × wirtgenii* [17]
5,6,7,4′-Tetrasubstituted flavones		
Scutellarein 7-*O*-glucuronide	*G. pubescens* [17]	
	*G. tetrahit* [17]	
Ladanetin		*G. ladanum* [18]
Ladanein		*G. ladanum* [18]
Salvigenin		*G. ladanum* subsp. *angustifolia* [13]
5,7,8,4′-Tetrasubstituted		
Isoscutellarein 7-*O*-(2″-*O*-allosyl)-glucoside		*G. ladanum* [17]
		*G. ladanum* subsp. *angustifolia* [17]
		*G. pyrenaica* [17]
		*G. segetum* [17]
		*G. × wirtgenii* [17]
Isoscutellarein 7-*O*-(2″-*O*-(6″’-*O*-acetyl)-allosyl)-glucoside		*G. ladanum* [17]
		*G. ladanum* subsp. *angustifolia* [17]
		*G. pyrenaica* [17]
		*G. segetum* [17]
		*G. × wirtgenii* [17]
Isoscutellarein 7-*O*-(2″-*O*-(6″’-*O*-acetyl)-allosyl-6″-*O*-acetyl)-		*G. ladanum* [17]
glucoside		*G. ladanum* subsp. *angustifolia* [17]
		*G. pyrenaica* [17]
		*G. segetum* [17]
		*G. × wirtgenii* [17]
Isoscutellarein 4′-methyl ester 7-*O*-(2″-*O*-(6″’-*O*-acetyl)-allosyl- 6″-*O*-acetyl)-glucoside		*G. ladanum* [19]
Galangustin		*G. ladanum* subsp. *angustifolia* [20]
5,7,3′,4′-Tetrasubstituted		
Luteolin		*G. segetum* [15]
Luteolin 7-*O*-glucuronude	*G. bifida* [17]	*G. ladanum* [17]
	*G. pubescens* [17]	*G. ladanum* subsp. *angustifolia* [17]
	*G. speciosa* [9]	*G. pyrenaica* [17]
	*G. tetrahit* [17]	*G. segetum* [17]
		*G. × wirtgenii* [17]
5,7,8,3′,4′-Pentasubstituted flavones		
8-Hydroxychrysoeriol 7-*O*-(2″-*O*-allosyl)-glucoside		*G. ladanum* subsp. *angustifolia* [21]
8-Hydroxychrysoeriol 7-*O*-(2″-*O*-(6″’-*O*-acetyl)-allosyl)-glucoside		*G. ladanum* subsp. *angustifolia* [21]
8-Hydroxychrysoeriol 7-*O*-(2″-*O*-(6″’-*O*-acetyl)-allosyl- 6″-*O*-acetyl)-glucoside		*G. ladanum* subsp. *angustifolia* [21]
Hypolaetin 7-*O*-(2″-*O*-allosyl)-glucoside		*G. ladanum* subsp. *angustifolia* [17]
Hypolaetin 7-*O*-(2″-*O*-(6″’-*O*-acetyl)-allosyl)-glucoside		*G. ladanum* [17]
		*G. ladanum* subsp. *angustifolia* [17]
		*G. pyrenaica* [17]
		*G. segetum* [17]
		*G. × wirtgenii* [17]
Hypolaetin 7-*O*-(2″-*O*-(6″’-*O*-acetyl)-allosyl- 6″-*O*-acetyl)-glucoside		*G. ladanum* [17]
		*G. ladanum* subsp. *angustifolia* [17]
		*G. segetum* [17]
Hypolaetin 4′-methyl ester 7-*O*-(2″-*O*-allosyl)-glucoside		*G. ladanum* [17]
		*G. ladanum* subsp. *angustifolia* [17]
		*G. segetum* [17]
Hypolaetin 4′-methyl ester 7-*O*-(2″-*O*-(6″’-*O*-acetyl)-allosyl)-		*G. ladanum* [17]
glucoside		*G. ladanum* subsp. *angustifolia* [17]
		*G. pyrenaica* [17]
		*G. segetum* [17]
Hypolaetin 4′-methyl ester 7-*O*-(2″-*O*-(6″’-*O*-acetyl)-allosyl-		*G. ladanum* [17]
6″-*O*-acetyl)-glucoside		*G. ladanum* subsp. *angustifolia* [17]
		*G. pyrenaica* [17]
		*G. segetum* [17]
		*G. × wirtgenii* [17]
Various		
Fatty acids	*G. bifida* [22,23,24,25]	
Acylglycerols	*G. bifida* [26]	
Essential oil	*G. bifida* [27]	
	*G. pubescens* [28]	
	*G. tetrahit* [28]	

**Table 3:** Content of selected compounds in *G. bifida* organs.

Compound	Content, mg/g of Dry Plant Weight ± S.D.			
	Leaves	Flowers	Stems	Roots
Iridoid glycosides				
Harpagide	11.35 ± 0.23	5.18 ± 0.11	9.37 ± 0.19	0.50 ± 0.01
Harpagide 8-*O*-acetate	25.69 ± 0.51	10.37 ± 0.20	18.53 ± 0.37	1.62 ± 0.03
Phenylethanoid glycosides				
Verbascoside	21.56 ± 0.51	18.98 ± 0.56	5.32 ± 0.14	2.63 ± 0.06
Isoverbascoside	14.88 ± 0.38	9.15 ± 0.23	2.15 ± 0.06	2.08 ± 0.05
Lavandulifolioside	10.21 ± 0.06	16.37 ± 0.40	8.79 ± 0.26	1.57 ± 0.04
Leucosceptoside A	9.37 ± 0.18	3.16 ± 0.06	1.75 ± 0.03	0.93 ± 0.02
Leonoside A	3.76 ± 0.07	1.58 ± 0.03	0.32 ± 0.01	0.11 ± 0.00
Leonoside B	1.60 ± 0.03	0.82 ± 0.02	0.14 ± 0.00	<0.01
Caffeoylquinic acids				
1-*O*-Caffeoylquinic acid	0.53 ± 0.01	0.18 ± 0.00	<0.01	<0.01
3-*O*-Caffeoylquinic acid	0.92 ± 0.02	2.61 ± 0.06	0.94 ± 0.02	<0.01
4-*O*-Caffeoylquinic acid	0.86 ± 0.02	0.25 ± 0.00	<0.01	<0.01
5-*O*-Caffeoylquinic acid	45.20 ± 1.31	12.97 ± 0.38	8.30 ± 0.25	2.90 ± 0.08
Flavone glycosides				
Luteolin 7-*O*-glucuronide	29.73 ± 0.59	39.63 ± 0.79	3.75 ± 0.07	0.15 ± 0.00
Apigenin 7-*O*-glucuronide	19.32 ± 0.37	1.93 ± 0.04	0.45 ± 0.01	0.14 ± 0.00
6-Hydroxyluteolin 7-*O*-glucuronide	2.63 ± 0.05	3.84 ± 0.07	0.35 ± 0.00	0.10 ± 0.00
Scutellarein 7-*O*-glucuronide	4.16 ± 0.08	5.22 ± 0.10	0.26 ± 0.00	0.11 ± 0.00
Luteolin 7-*O*-(6″-O-*p*-coumaroyl)-glucoside	11.79 ± 0.23	1.84 ± 0.03	0.27 ± 0.00	<0.01
Apigenin 7-*O*-(6″-O-*p*-coumaroyl)-glucoside	12.93 ± 0.25	1.02 ± 0.02	1.53 ± 0.03	<0.01
Total content				
Iridoid glucosides	37.04	15.55	27.90	2.12
Phenylethanoid glucosides	61.38	50.06	18.47	7.32
Caffeoylquinic acids	47.51	16.01	9.21	2.90
Flavone glycosides	80.56	53.48	6.61	0.50
Phenolic compounds	189.45	119.55	34.29	10.72

**Table 4:** Content of selected compounds in leaves of *G. bifida* from eight Siberian populations (P1–P8).

Compound	Content in Populations, mg/g of Dry Plant Weight ± S.D. (Variation Coefficient, %)							
	P1 (*n* = 21) ^a^	P2 (*n* = 30) ^a^	P3 (*n* = 34) ^a^	P4 (*n* = 28) ^a^	P5 (*n* = 18) ^a^	P6 (*n* = 25) ^a^	P7 (*n* = 20) ^a^	P8 (*n* = 17) ^a^
Iridoid glycosides								
Harpagide	8.57 ± 0.42 (4.9)	10.36 ± 0.61 (5.9)	14.69 ± 1.29 (8.8)	18.33 ± 1.41 (7.7)	2.95 ± 0.20 (6.8)	4.14 ± 0.23 (5.6)	2.16 ± 0.16 (7.4)	<0.01
Harpagide 8-*O*-acetate	14.53 ± 1.14 (7.9)	10.69 ± 1.10 (10.3)	11.82 ± 0.98 (8.3)	9.35 ± 1.09 (11.7)	24.52 ± 1.83 (7.5)	27.18 ± 1.47 (5.4)	31.82 ± 1.56 (4.9)	27.53 ± 1.73 (6.3)
Phenylethanoid glycosides								
Verbascoside	10.32 ± 0.64 (6.2)	8.54 ± 0.79 (9.3)	5.63 ± 0.47 (8.3)	5.07 ± 0.52 (10.3)	20.67 ± 1.01 (4.9)	25.16 ± 1.24 (4.9)	22.67 ± 1.79 (7.9)	27.59 ± 2.23 (8.1)
Isoverbascoside	<0.01	<0.01	<0.01	<0.01	15.02 ± 0.85 (5.7)	12.76 ± 0.51 (4.0)	17.73 ± 1.98 (11.2)	18.67 ± 1.56 (8.4)
Lavandulifolioside	1.53 ± 0.09 (5.9)	0.94 ± 0.08 (8.5)	0.27 ± 0.03 (11.1)	0.59 ± 0.05 (8.5)	10.86 ± 0.67 (6.2)	9.82 ± 0.81 (8.2)	11.67 ± 0.57 (4.9)	10.33 ± 0.60 (5.8)
Leucosceptoside A	0.56 ± 0.05 (8.9)	0.42 ± 0.04 (9.5)	0.31 ± 0.03 (9.7)	0.12 ± 0.01 (8.3)	9.95 ± 0.91 (9.2)	10.53 ± 0.39 (3.7)	8.64 ± 0.51 (5.9)	9.37 ± 0.62 (6.6)
Leonoside A	0.32 ± 0.03 (9.4)	0.43 ± 0.03 (7.0)	<0.01	<0.01	3.09 ± 0.33 (10.7)	4.29 ± 0.27 (6.3)	3.52 ± 0.15 (4.3)	2.11 ± 0.10 (4.7)
Leonoside B	<0.01	<0.01	<0.01	1.72 ± 0.17 (9.9)	1.57 ± 0.14 (8.9)	1.43 ± 0.11 (7.7)	1.93 ± 0.09 (4.7)	1.72 ± 0.17 (9.9)
Caffeoylquinic acids								
1-*O*-Caffeoylquinic acid	<0.01	<0.01	<0.01	<0.01	0.62 ± 0.03 (4.8)	0.27 ± 0.02 (7.4)	<0.01	<0.01
3-*O*-Caffeoylquinic acid	0.94 ± 0.08 (8.5)	0.52 ± 0.05 (9.6)	0.37 ± 0.03 (8.1)	<0.01	1.02 ± 0.05 (4.9)	0.53 ± 0.03 (5.7)	0.47 ± 0.03 (6.4)	<0.01
4-*O*-Caffeoylquinic acid	0.22 ± 0.02 (9.1)	<0.01	<0.01	<0.01	0.73 ± 0.05 (6.8)	0.56 ± 0.05 (8.9)	0.18 ± 0.02 (11.1)	<0.01
5-*O*-Caffeoylquinic acid	12.67 ± 1.06 (8.4)	11.73 ± 1.45 (12.4)	9.69 ± 0.56 (5.8)	5.33 ± 0.55 (10.3)	42.53 ± 1.65 (3.9)	41.75 ± 3.95 (9.5)	36.18 ± 4.23 (11.7)	35.02 ± 2.94 (8.4)
Flavone glycosides								
Luteolin 7-*O*-glucuronide	32.59 ± 1.89 (5.8)	46.14 ± 2.26 (4.9)	45.53 ± 3.82 (8.3)	42.76 ± 3.12 (7.3)	27.63 ± 2.32 (8.4)	25.85 ± 1.47 (5.7)	22.63 ± 2.47 (10.9)	19.07 ± 0.95 (5.0)
Apigenin 7-*O*-glucuronide	22.73 ± 1.45 (6.4)	25.82 ± 1.49 (5.8)	27.59 ± 1.71 (6.2)	25.07 ± 1.43 (5.7)	17.67 ± 2.19 (12.4)	15.72 ± 1.07 (6.8)	12.04 ± 1.01 (8.4)	10.35 ± 0.51 (4.9)
6-Hydroxyluteolin 7-*O*-glucuronide	3.67 ± 0.21 (5.7)	4.57 ± 0.18 (3.9)	4.96 ± 0.35 (7.1)	4.50 ± 0.35 (7.8)	2.90 ± 0.22 (7.6)	1.27 ± 0.10 (7.9)	0.95 ± 0.06 (6.3)	1.11 ± 0.10 (9.0)
Scutellarein 7-*O*-glucuronide	2.75 ± 0.18 (6.5)	3.57 ± 0.17 (4.8)	4.18 ± 0.22 (5.3)	4.09 ± 0.39 (9.5)	3.84 ± 0.23 (6.0)	2.04 ± 0.11 (5.3)	1.57 ± 0.09 (5.7)	2.47 ± 0.23 (9.3)
Luteolin 7-*O*-(6″-O-*p*-coumaroyl)-glucoside	0.52 ± 0.05 (9.6)	0.37 ± 0.03 (8.1)	0.11 ± 0.01 (9.1)	<0.01	12.04 ± 0.89 (7.4)	12.64 ± 0.87 (6.9)	27.35 ± 2.19 (8.0)	17.36 ± 1.02 (5.9)
Apigenin 7-*O*-(6″-O-*p*-coumaroyl)-glucoside	<0.01	<0.01	<0.01	<0.01	14.07 ± 0.73 (5.2)	17.53 ± 0.86 (4.9)	29.11 ± 1.14 (3.9)	20.63 ± 1.44 (7.0)
Total content								
Iridoid glucosides	23.10	21.05	26.51	27.68	25.47	31.32	33.98	27.53
Phenylethanoid glucosides	12.73	10.33	6.21	7.50	61.16	63.99	66.16	69.79
Saffeoylquinic acids	13.83	12.25	10.06	5.33	44.90	43.11	36.83	35.02
Non-acylated flavone glycosides	61.74	80.05	82.26	76.42	52.04	44.88	37.19	33.00
Acylated flavone glycosides	0.52	0.37	0.11	<0.01	26.11	30.17	56.46	37.99
Flavone glycosides	62.26	80.42	82.37	76.42	78.15	75.05	93.65	70.99

^a^*n*—number of plant samples used for analysis.

**Table 5:** Antioxidant activity of *G. bifida* extracts in six assays ^a^, in μM trolox-eq./g of dry weight ± S.D.

Extract No	DPPH	ABTS	SSA	FRAP	ORAC	CBA
P1	286.6 ± 5.7 ^a^	293.4 ± 8.8 ^g^	182.4 ± 7.2 ^y^	103.9 ± 4.1 ^l^	253.0 ± 7.5 ^p^	298.3 ± 14.9 ^t^
P2	347.1 ± 6.9 ^c^	326.2 ± 9.7 ^h^	193.6 ± 7.7 ^y^	115.2 ± 4.6 ^lm^	296.1 ± 8.9 ^p^	343.1 ± 17.1 ^u^
P3	353.2 ± 8.9 ^c^	373.8 ± 11.2 ^i^	202.8 ± 8.1 ^y^	125.9 ± 5.0 ^m^	315.2 ± 9.4 ^q^	374.1 ± 18.7 ^v^
P4	302.1 ± 6.0 ^b^	325.6 ± 9.7 ^h^	189.4 ± 7.5 ^y^	109.6 ± 4.4 ^l^	310.6 ± 9.3 ^pq^	357.2 ± 17.8 ^uv^
P5	533.8 ± 10.6 ^d^	618.2 ± 18.5 ^j^	294.7 ± 11.8 ^z^	306.2 ± 12.2 ^n^	576.3 ± 17.2 ^r^	657.6 ± 32.8 ^w^
P6	587.1 ± 11.7 ^e^	624.3 ± 18.7 ^j^	326.8 ± 12.9 ^z^	312.4 ± 12.4 ^n^	582.9 ± 17.4 ^r^	699.2 ± 34.9 ^w^
P7	632.4 ± 12.5 ^f^	693.0 ± 19.5 ^k^	363.7 ± 14.5 ^ã^	329.1 ± 12.9 ^no^	631.0 ± 18.3 ^s^	734.8 ± 36.2 ^x^
P8	604.4 ± 12.0 ^e^	646.2 ± 19.2 ^j^	318.2 ± 12.7 ^z^	361.2 ± 14.0 ^o^	596.7 ± 17.9 ^rs^	701.4 ± 35.0 ^wx^

^a^ DPPH—scavenging capacity against 2,2-diphenyl-1-picrylhydrazyl radical; ABTS—scavenging capacity against 2,2′-azino-bis(3-ethylbenzothiazoline-6-sulfonic acid) cation radical; SSA—scavenging capacity against superoxide radical; FRAP—ferric reducing antioxidant power; ORAC—oxygen radical absorbance capacity; CBA—carotene bleaching assay. Averages ± standard deviation (S.D.) were obtained from five different experiments. Values with different letters (^a–z^, ã) indicate statistically significant differences among groups at *p* < 0.05 by one-way ANOVA.

**Table 6:** Regression coefficients (*r*^2^) of “antioxidant activity–compound content” relationships.

Compounds	DPPH	ABTS	SSA	FRAP	ORAC	CBA
Iridoid glucosides	0.1726	0.1671	0.1726	0.0948	0.1488	0.1498
Phenylethanoid glucosides	0.9565	0.9437	0.9349	0.9828	0.9477	0.9540
Caffeoylquinic acids	0.8935	0.8880	0.8722	0.8960	0.8861	0.8954
Non-acylated flavone glycosides	0.6792	0.7288	0.7124	0.7862	0.7454	0.7352
Acylated flavone glycosides	0.9325	0.9237	0.9486	0.9109	0.9114	0.9102
Flavone glycosides	0.4458	0.5264	0.4333	0.5797	0.5868	0.5631

**Table 7:** Detailed information about *Galeopsis bifida* populations P1–P8.

Number	Collection Place	Population Area, km^2^	Collection Date	Coordinates	Height (m a.s.l.)	Voucher Specimens No
P1	Kizhinga, Kizhinginskii District, Republic Buryatia	2.5	20.VI.2019	51°47′44.0″ N, 109°52′24.6″ E	670	BU/LAM-0619/59–114
P2	Babushkin, Kabanskii District, Republic Buryatia	2.8	20.VI.2019	51°41′18.3″ N, 105°50′39.4″ E	660	BU/LAM-0619/63–127
P3	Tsakir, Zakamenskii District, Republic Buryatia	1.7	20.VI.2019	50°24′54.7″ N, 103°34′42.0″ E	1100	BU/LAM-0619/76–139
P4	Tamir, Kyakhtinskii District, Republic Buryatia	0.9	20.VI.2019	50°12′51.8″ N, 107°25′34.7″ E	1150	BU/LAM-0619/79–146
P5	Vilyuisk, Viluiskii Ulus, Republic Sakha (Yakutia)	0.5	20.VI.2019	63°43′07.3″ N, 121°38′55.9″ E	110	YA/LAM-0619/269–418
P6	Yakutsk, Republic Sakha (Yakutia)	0.4	20.VI.2019	62°00′51.1″ N, 129°38′06.6″ E	100	YA/LAM-0619/273–425
P7	Ust-Nera, Oymyakonskii Ulus, Republic Sakha (Yakutia)	0.9	20.VI.2019	64°32′23.6″ N, 143°14′49.4″ E	690	YA/LAM-0619/293–453
P8	Verkhoyansk, Verkhoyanskii Ulus, Republic Sakha (Yakutia)	0.2	20.VI.2019	67°27′53.3″ N, 133°24′37.0″ E	400	YA/LAM-0619/299–457

## Supplementary Materials

The following are available online at https://www.mdpi.com/2223-7747/9/11/1555/s1, Table S1: Ultraviolet spectral patterns of compounds found in *Galeopsis bifida*, Table S2: Content of selected compounds in extracts of *G. bifida* from eight Siberian populations, Table S3: Reference standards used for the qualitative and quantitative analysis by HPLC-PAD-ESI-tQ-MS, Table S4: Regression equations, correlation coefficients, standard deviation, limits of detection, limits of quantification and linear ranges for 17 reference standards used in HPLC-MS quantification.
