# Supplementary material for: Synanthropic Plants as an Underestimated Source of Bioactive Phytochemicals: A Case of Galeopsis bifida (Lamiaceae)
Source: Plants (Basel). 2020 Nov 12;9(11):1555. doi: 10.3390/plants9111555 (PMC7696744; doi:10.3390/plants9111555)
Supplement: Supplementary file 1 [file plants-09-01555-s001.pdf]

plants

Supplementary materials

Synanthropic Plants as an Underestimated Source of Bioactive Phytochemicals: A case of *Galeopsis bifida* (Lamiaceae)

Daniil N. Olennikov

Laboratory of Medical and Biological Research, Institute of General and Experimental Biology, Siberian Division, Russian Academy of Science, 6 Sakhyanovoy Street, Ulan-Ude 670047, Russia; olennikovdn@mail.ru; Tel.: +7-9021-600-627

Received: 29 October 2020; Accepted: 11 November 2020; Published: date

Content

Table S1. Ultraviolet spectral patterns of compounds found in *Galeopsis bifida*.

Table S2. Content of selected compounds in extracts of *G. bifida* from eight Siberian populations.

Table S3. Reference standards used for the qualitative and quantitative analysis by HPLC-PAD-ESI-tQ-MS.

Table S4. Regression equations, correlation coefficients, standard deviation, limits of detection, limits of quantification and linear ranges for 17 reference standards used in HPLC-MS quantification.

Table S1. Ultraviolet spectral patterns of compounds found in *Galeopsis bifida*.

Name of UV-pattern	Group of compound	λ_{max}, nm
AG	Apigenin glycoside	268 (±2), 332 (±3)
AGC	Apigenin glycoside acylated with *p*-coumaric acid	267 (±2), 315 (±3)
CQA	Caffeoylquinic acid	295 (±1), 324 (±1)
HLG	6-Hydroxyluteolin glycoside	352 sh (±1), 280 (±2), 343 (±3)
IG	Iridoid glycoside	203 (±5)
LG	Luteolin glucoside	255 (±2), 268 sh (±2), 345 (±3)
LGC	Luteolin glycoside acylated with *p*-coumaric acid	256 (±1), 273 sh (±2), 333 (±3)
PEG	Phenylethanoid glycoside	297 (±1), 330 (±2)
SG	Scutellarein glycoside	283 (±2), 337 (±2)
SGC	Scutellarein glycoside acylated with *p*-coumaric acid	284 (±2), 325 (±4)

sh.—sholder

Table S2. Content of selected compounds in extracts of *G. bifida* from eight Siberian populations (P1–P8)

**Compound**	**Content in dry extract, mg/g of dry extract weight ± S.D.**			
	P1	P2	P3	P4
Iridoid glycosides				
Harpagide	29.14 ± 0.58	42.48 ± 0.85	58.76 ± 1.17	58.65 ± 1.19
Harpagide 8-*O*-acetate	49.40 ± 0.99	42.76 ± 0.91	47.28 ± 94.56	31.79 ± 0.63
Phenylethanoid glycosides				
Verbascoside	35.09 ± 0.70	34.16 ± 0.68	22.52 ± 0.45	17.23 ± 0.34
Isoverbascoside	<0.01	<0.01	<0.01	<0.01
Lavandulifolioside	5.20 ± 0.10	3.76 ± 0.07	1.08 ± 0.02	2.01 ± 0.04
Leucosceptoside A	1.92 ± 0.04	1.69 ± 0.03	1.24 ± 0.02	0.41 ± 0.01
Leonoside A	1.09 ± 0.02	1.72 ± 0.03	<0.01	<0.01
Leonoside B	<0.01	<0.01	<0.01	5.85 ± 0.12
Caffeoylquinic acids				
1-*O*-Caffeoylquinic acid	<0.01	<0.01	<0.01	<0.01
3-*O*-Caffeoylquinic acid	3.20 ± 0.06	2.08 ± 0.04	1.48 ± 0.03	<0.01
4-*O*-Caffeoylquinic acid	0.74 ± 0.02	<0.01	<0.01	<0.01
5-*O*-Caffeoylquinic acid	43.08 ± 0.86	46.92 ± 0.93	38.76 ± 0.78	18.12 ± 0.37
Flavone glycosides				
Luteolin 7-*O*-glucuronide	110.81 ± 2.21	184.56 ± 3.69	182.12 ± 3.64	145.38 ± 2.91
Apigenin 7-*O*-glucuronide	77.28 ± 1.54	103.28 ± 2.06	110.36 ± 2.21	85.23 ± 1.70
6-Hydroxyluteolin 7-*O*-glucuronide	12.48 ± 0.25	18.28 ± 0.36	19.84 ± 39.68	15.30 ± 0.31
Scutellarein 7-*O*-glucuronide	9.35 ± 0.19	14.28 ± 0.28	16.72 ± 0.33	14.01 ± 0.29
Luteolin 7-*O*-(6′′-O-*p*-coumaroyl)-glucoside	1.76 ± 0.03	1.48 ± 0.03	0.44 ± 0.01	<0.01
Apigenin 7-*O*-(6′′-O-*p*-coumaroyl)-glucoside	<0.01	<0.01	<0.01	<0.01
Total content				
Iridoid glucosides	58.54	85.24	106.04	90.44
Phenylethanoid glucosides	43.30	41.33	24.84	25.50
Caffeoylquinic acids	47.02	49.00	40.24	18.12
Non-acylated flavone glycosides	209.92	320.40	329.04	259.92
Acylated flavone glycosides	1.76	1.48	0.44	<0.01
Flavone glycosides	211.68	321.88	329.48	259.92

Table S2. *Cont.*

Compound	Content in dry extract, mg/g of dry extract weight ± S.D.			
	P5	P6	P7	P8
Iridoid glycosides				
Harpagide	8.85 ± 0.17	11.59 ± 0.23	6.91 ± 0.14	<0.01
Harpagide 8-*O*-acetate	73.56 ± 1.47	76.10 ± 1.52	101.82 ± 2.04	90.85 ± 1.81
Phenylethanoid glycosides				
Verbascoside	62.01 ± 1.24	70.45 ± 1.41	72.54 ± 1.45	91.05 ± 1.85
Isoverbascoside	35.73 ± 0.71	45.06 ± 0.90	56.74 ± 1.14	61.61 ± 1.23
Lavandulifolioside	29.46 ± 0.59	32.58 ± 0.65	37.34 ± 0.75	34.08 ± 0.68
Leucosceptoside A	29.85 ± 0.59	31.59 ± 0.63	27.65 ± 0.55	30.92 ± 0.62
Leonoside A	9.27 ± 0.18	12.87 ± 0.25	11.26 ± 0.23	6.96 ± 0.14
Leonoside B	4.71 ± 0.09	4.29 ± 0.08	6.17 ± 0.12	5.67 ± 0.11
Caffeoylquinic acids				
1-*O*-Caffeoylquinic acid	0.81 ± 0.02	1.86 ± 0.03	<0.01	<0.01
3-*O*-Caffeoylquinic acid	1.59 ± 0.03	3.06 ± 0.06	1.50 ± 0.03	<0.01
4-*O*-Caffeoylquinic acid	1.62 ± 0.03	2.19 ± 0.04	0.57 ± 0.01	<0.01
5-*O*-Caffeoylquinic acid	116.90 ± 2.33	127.59 ± 2.55	115.78 ± 2.31	112.57 ± 2.24
Flavone glycosides				
Luteolin 7-*O*-glucuronide	72.38 ± 1.45	82.89 ± 1.65	72.42 ± 1.45	62.93 ± 1.25
Apigenin 7-*O*-glucuronide	44.01 ± 0.88	53.01 ± 1.07	38.52 ± 0.77	34.15 ± 0.68
6-Hydroxyluteolin 7-*O*-glucuronide	3.55 ± 0.07	8.70 ± 0.17	3.04 ± 0.06	3.66 ± 0.07
Scutellarein 7-*O*-glucuronide	2.71 ± 0.11	11.52 ± 0.23	5.02 ± 0.10	8.15 ± 0.16
Luteolin 7-*O*-(6′′-O-*p*-coumaroyl)-glucoside	35.39 ± 0.70	36.12 ± 0.72	57.52 ± 1.15	57.29 ± 1.12
Apigenin 7-*O*-(6′′-O-*p*-coumaroyl)-glucoside	42.21 ± 0.84	49.08 ± 0.98	93.15 ± 1.86	68.08 ± 1.36
Total content				
Iridoid glucosides	82.41	87.69	108.73	90.85
Phenylethanoid glucosides	171.03	196.84	211.17	230.29
Caffeoylquinic acids	120.92	134.70	117.85	112.57
Non-acylated flavone glycosides	122.65	156.12	119.00	108.89
Acylated flavone glycosides	77.60	85.20	150.67	125.37
Flavone glycosides	200.25	241.32	269.67	234.26

Table S3. Reference standards used for the qualitative and quantitative analysis by HPLC-PAD-ESI-tQ-MS.

Standard, formula [a]	Purity, %	Manufacturer (Cat. no) [b]	Quantified substance (no in Table 2)
Iridoids			
Ajugol (=leonuride) 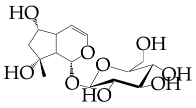	≥ 98	ChemFaces (CFN90759)	-
Ajugoside 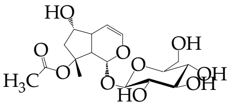	≥ 88	MedKoo (592335)	-
Harpagide 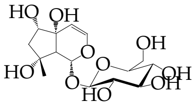	≥ 95	Sigma (PHL89703)	Harpagide (**3**)
Harpagide 8-*O*-acetate 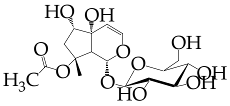	≥ 95	Sigma (PHL82700)	Harpagide 8-*O*-acetate (**15**)
Secologanin 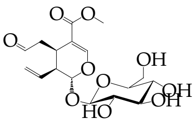	≥ 88	Sigma (50741)	-
Reptoside 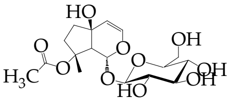	≥ 95	Toronto (R144658)	-
Phenylethanoid glycosides			
Isoverbascoside (=isoacteoside) 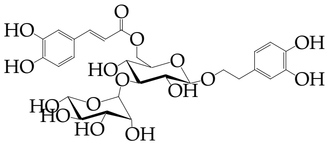	≥ 90	Sigma (PHL89232)	Isoverbascoside (**45**)
Lavandulifolioside 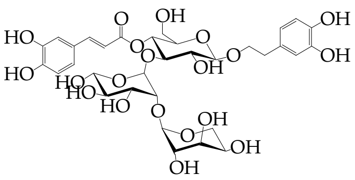	≥ 90	Lab collection/isolated from *Leonurus deminutus* [55]	-

Table S3. *Cont.*

Standard, formula [a]	Purity, %	Manufacturer (Cat. no) [b]	Quantified substance (no in Table 2)
Leonoside A<br>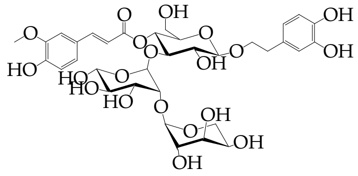	≥ 98	MedKoo (598584)	Leonoside A (**43**)
Leonoside B<br>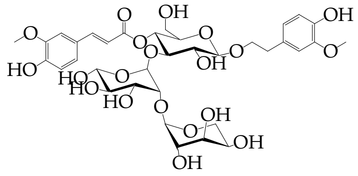	≥ 98	MedKoo (598583)	Leonoside B (**47**)
Leucosceptoside A<br>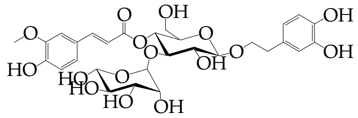	≥ 98	ChemFaces (CFN89166)	Leucosceptoside A (**46**)
Martynoside<br>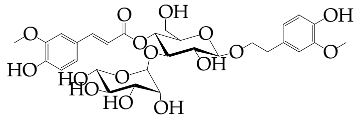	≥ 98	ChemFaces (CFN97159)	-
Verbascoside (=acteoside)<br>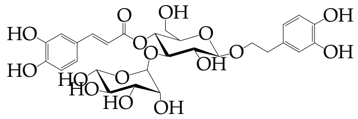	≥ 99	Sigma (V4015)	Verbascoside (**42**)<br>Lavandulifolioside (**38**)
		Hydroxycinnamates	
1-*O*-Caffeoylquinic acid<br>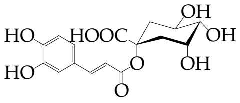	≥ 98	ChemFaces (CFN99121)	1-*O*-Caffeoylquinic acid (**65**)
3-*O*-Caffeoylquinic acid<br>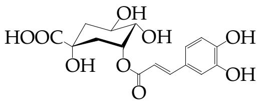	≥ 95	Sigma (C3878)	3-*O*-Caffeoylquinic acid (**69**)
4-*O*-Caffeoylquinic acid<br>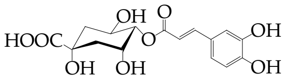	≥ 98	Sigma (65969)	4-*O*-Caffeoylquinic acid (**66**)

Table S3. *Cont.*

**Standard, formula** [a]	**Purity, %**	**Manufacturer (Cat. no)** [b]	**Quantified substance (no in Table 2)**
5-*O*-Caffeoylquinic acid 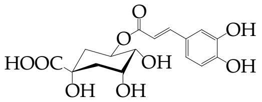	≥ 98	Sigma (94419)	5-*O*-Caffeoylquinic acid (**68**)
Phaselic acid 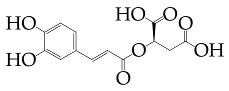	≥ 90	Lab collection/isolated from *Leonurus deminutus* [55]	-
		Flavone glycosides	
Apigenin 7-*O*-glucoside (=cosmosiin) 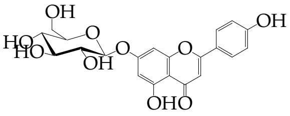	≥ 99	Extrasynthese (1004 S)	-
Apigenin 7-*O*-glucuronide 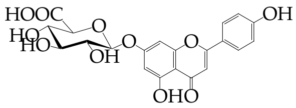	≥ 98	ChemFaces (CFN98500)	Apigenin 7-*O*-glucuronide (**78**)
Apigenin 7-*O*-(6′′-*O*-*p*-coumaroyl)-glucoside 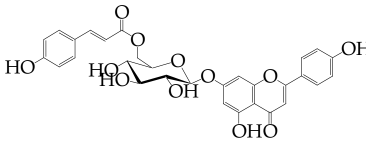	≥ 95	VILAR (071018)	Apigenin 7-*O*-(6′′-*O*-*p*-coumaroyl)-glucoside (**86**)
6-Hydroxyluteolin 7-*O*-glucoside 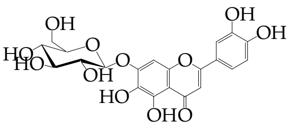	≥ 98	ChemFaces (CFN91094)	6-Hydroxyluteolin 7-*O*-glucuronide (**73**)
6-Hydroxyluteolin 7-*O*-glucuronide 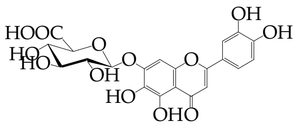	≥ 90	Lab collection/isolated from *Rhaponticum uniflorum* [57]	-
Luteolin 7-*O*-glucoside (=cynaroside) 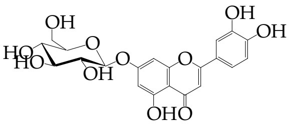	≥ 98	Extrasynthese (1126 S)	-

Table S3. *Cont.*

**Standard, formula** [a]	**Purity, %**	**Manufacturer (Cat. no)** [b]	**Quantified substance (no in Table 2)**
Luteolin 7-*O*-glucuronide 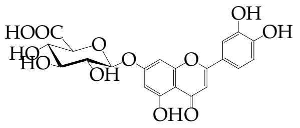	≥ 98	ChemFaces (CFN98512)	Luteolin 7-*O*-glucuronide (**74**)
Luteolin 7-*O*-(6′′-*O*-*p*-coumaroyl)-glucoside 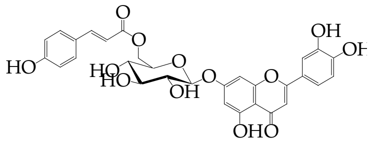	≥ 92	VILAR (230216)	Luteolin 7-*O*-(6′′-*O*-*p*-coumaroyl)-glucoside (**84**)
Scutellarein 7-*O*-glucuronide (=scutellarin) 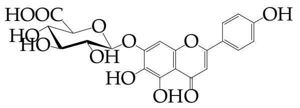	≥ 98	ChemFaces (CFN99112)	Scitellarein 7-*O*-glucuronide (**76**)
		Internal standards	
3,5-Di-*O*-Feruloylquinic acid 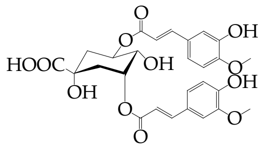	≥ 95	Lab collection/isolated from *Panax vietnamensis* [105]	-
Scopoletin 7-*O*-neohesperidoside 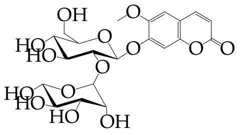	≥ 95	Lab collection/isolated from *Calendula officinalis* [106]	-
Trifloroside 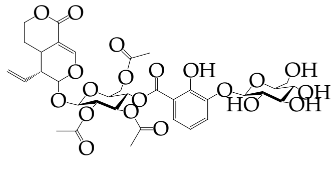	≥ 98	ChemFaces (CFN96606)	-

Manufacturers list: ChemFaces—ChemFaces (Wuhan, Hubei, PRC); Extrasynthese—Extrasynthese (Lyon, France); MedKoo—MedKoo Biosciences Inc. (Morrisville, NC, USA); Sigma—Sigma-Aldrich (St. Louis, MO, USA); Toronto—Toronto Research Chemicals (North York, ON, Canada); VILAR—VILAR Corp. (Moscow, Russia).

Table S4. Regression equations, correlation coefficients (r^2), standard deviation (S_{YX}), limits of detection (LOD), limits of quantification (LOQ) and linear ranges for 17 reference standards used for HPLC-MS quantification.

**Compound**	**Ionization** [a]	**CE** [b] **(eV)**	**Regression equation** [c]		r^2	S_{YX}	**LOD/ LOQ (μg/mL)**	**Linear range (μg/mL)**
			a	$b \cdot 10^6$				
Harpagide	P	+20	6.375	-0.146	0.9982	$1.09 \cdot 10^{-2}$	0.007/0.02	0.02–300.0
Harpagide 8-*O*-acetate	P	+20	7.351	-0.110	0.9979	$0.93 \cdot 10^{-2}$	0.004/0.01	0.01–300.0
Verbascoside	N	-25	2.733	-0.637	0.9991	$0.52 \cdot 10^{-2}$	0.006/0.02	0.02–250.0
Isoverbascoside	N	-25	2.536	-0.473	0.9962	$0.64 \cdot 10^{-2}$	0.008/0.03	0.03–250.0
Leucosceptoside A	N	-25	3.022	-0.769	0.9990	$1.02 \cdot 10^{-2}$	0.011/0.03	0.03–250.0
Leonoside A	N	-25	1.933	-0.562	0.9954	$0.83 \cdot 10^{-2}$	0.014/0.04	0.04–250.0
Leonoside B	N	-27	1.706	-0.485	0.9963	$0.79 \cdot 10^{-2}$	0.015/0.05	0.05–250.0
1-*O*-Caffeoylquinic acid	N	-15	2.539	-1.236	0.9994	$0.45 \cdot 10^{-2}$	0.006/0.02	0.02–300.0
3-*O*-Caffeoylquinic acid	N	-15	2.417	-1.567	0.9994	$0.40 \cdot 10^{-2}$	0.005/0.02	0.02–300.0
4-*O*-Caffeoylquinic acid	N	-15	2.736	-1.069	0.9996	$0.51 \cdot 10^{-2}$	0.006/0.02	0.02–300.0
5-*O*-Caffeoylquinic acid	N	-15	2.902	-1.418	0.9998	$0.39 \cdot 10^{-2}$	0.004/0.01	0.02–300.0
Apigenin 7-*O*-glucuronide	N	-20	5.802	-0.804	0.9990	$1.14 \cdot 10^{-2}$	0.007/0.02	0.02–500.0
Luteolin 7-*O*-glucuronide	N	-20	7.064	-1.533	0.9992	$1.92 \cdot 10^{-2}$	0.009/0.03	0.03–500.0
6-Hydroxyluteolin 7-*O*-glucoside	N	-20	7.833	-1.442	0.9984	$2.63 \cdot 10^{-2}$	0.011/0.03	0.04–500.0
Scutellarein 7-*O*-glucuronide	N	-30	5.206	-1.407	0.9992	$1.52 \cdot 10^{-2}$	0.010/0.03	0.03–500.0
Apigenin 7-*O*-(6′′-*O*-*p*-coumaroyl)-glucoside	N	-30	5.534	-0.705	0.9953	$2.01 \cdot 10^{-2}$	0.012/0.04	0.04–250.0
Luteolin 7-*O*-(6′′-*O*-*p*-coumaroyl)-glucoside	N	-25	7.804	-1.202	0.9944	$3.04 \cdot 10^{-2}$	0.012/0.04	0.04–250.0

[a] Ionization mode : [N]—negative; [P]—positive. [b] CE—collision energy. [c] Regression equation: $y = a \cdot x + b$.
